# Are Sexual Desire and Sociosexual Orientation Related to Men’s Salivary Steroid Hormones?

**DOI:** 10.1007/s40750-020-00148-y

**Published:** 2020-08-24

**Authors:** Julia Stern, Konstantina Karastoyanova, Michal Kandrik, Jaimie Torrance, Amanda C. Hahn, Iris Holzleitner, Lisa M. DeBruine, Benedict C. Jones

**Affiliations:** 1 Department of Psychology & Leibniz Science Campus Primate Cognition, University of Goettingen, Goettingen, Germany; 2 School of Social Work and Social Policy, University of Strathclyde, Glasgow, UK; 3 Department of Experimental and Applied Psychology, VU Amsterdam, Amsterdam, Netherlands; 4 Institute of Neuroscience & Psychology, University of Glasgow, Glasgow, Scotland; 5 Department of Psychology, Humboldt State University, Arcata, CA USA; 6 School of Psychological Sciences and Health, University of Strathclyde, Glasgow, UK

**Keywords:** Sexual desire, Sociosexual orientation, Testosterone, Cortisol, Dual Hormone Hypothesis

## Abstract

**Objective:**

Although it is widely assumed that men’s sexual desire and interest in casual sex (i.e., sociosexual orientation) are linked to steroid hormone levels, evidence for such associations is mixed.

**Methods:**

We tested for both longitudinal and cross-sectional relationships between salivary testosterone, cortisol, reported sexual desire and sociosexuality in a sample of 61 young adult men, each of whom was tested weekly on up to five occasions.

**Results:**

Longitudinal analyses showed no clear relationships between steroid hormones and self-reported sexual desire or sociosexual orientation. Cross-sectional analyses showed no significant associations between average hormone levels and self-reported sexual desire. However, some aspects of sociosexuality, most notably desire for casual sex, were related to men’s average hormone levels. Men with higher average testosterone reported greater desire for casual sex, but only if they also had relatively low average cortisol levels.

**Conclusions:**

Our results support a Dual Hormone account of men’s sociosexuality, in which the *combined* effects of testosterone and cortisol predict the extent of men’s interest in casual sex. However, we did not detect compelling evidence for an association of within-subject hormone shifts and sexual desire or sociosexual orientation.

**Electronic supplementary material:**

The online version of this article (10.1007/s40750-020-00148-y) contains supplementary material, which is available to authorized users.

## Introduction

Inspired by findings from non-human primates (reviewed in Roney 2018), many recent studies have used longitudinal designs to investigate the hormonal correlates of within-woman changes in both sexual desire (solitary sexual desire, which reflects the desire for sexual behavior with oneself, or dyadic sexual desire, the desire for engaging in sex with a partner) and sociosexual orientation (the openness to uncommitted sex, which can be subdivided into sociosexual behavior, desire and attitudes). Results on this topic have been somewhat mixed, however. Most studies suggest a general increase in sexual desire when estradiol is high and/or progesterone is low (Arslan et al. 2020; Jones et al. 2018; Roney and Simmons 2013; 2016; Shirazi et al. 2019a). By contrast, other work has reported that openness to uncommitted sexual relationships, but not general sexual desire, increases when estradiol is high (Shirazi et al. 2019b) or that desire for sex with women’s primary partner increases when progesterone is high (Grebe et al. 2016).

Somewhat surprisingly, there have been far fewer longitudinal studies of the hormonal correlates of within-man changes in sexual desire and sociosexual orientation. Steroid hormones in men do not change systematically from day-to-day or week-to-week, as compared to hormonal changes across women’s ovulatory cycle, but rather from morning to evening (Papacosta and Nassis 2011) or seasonally (Stanton et al. 2011). However, testosterone, men’s primary sex hormone, is highly variable and reactive in response to different social cues. For example, testosterone has previously been reported to increase as a reaction to winning a competition (Geniole et al. 2017), social interactions with young women (Roney et al. 2009), watching or participating in sexual activity (Escasa et al. 2011), or to decrease when sleep deprivated (Cote et al. 2013). Moreover, prescriptions for testosterone to treat symptoms of sexual dysfunction (e.g. libido) are common (e.g. Petering and Brooks 2017), relying on the assumption that an increase in within-men’s testosterone levels is related to a higher libido (e.g. increasing sexual desire). While some studies report a small increase in men’s libido after being treated with testosterone (e.g. Cunningham et al. 2016), overall evidence whether testosterone treatments really lead to clinically significant benefits is still mixed (Petering and Brooks 2017). In addition, it remains unclear whether natural changes in testosterone are related to sexual desire or sociosexual orientation as well. Importantly, the only study that reports longitudinal results (within-subjects changes) in a non-clinical sample does not find compelling evidence for a link between within-men changes in sociosexual orientation and testosterone (Gettler et al. 2019).

There are somewhat more studies that investigated links between testosterone and between-men’s sexual desire or sociosexual orientation. However, results of these cross-sectional (between-subjects) studies are fairly mixed. Whereas positive associations between testosterone and solitary, but not dyadic, sexual desire have been reported in one study (van Anders and Dunn 2009), no associations between testosterone and solitary or dyadic sexual desire were observed in two other studies (van Anders et al. 2007; van Anders 2012). Similarly, one study reported that sociosexual desire, but not sociosexual behavior or attitudes, was positively correlated with between-men’s testosterone (Edelstein et al. 2011), two studies found no compelling evidence for correlations between testosterone and sociosexual orientation (Kordsmeyer et al. 2018; van Anders et al. 2007), and one study found that testosterone was positively correlated with sociosexual desire, but negatively correlated with sociosexual behavior (Puts et al. 2015). For an overview of previous studies, including their sample sizes and results, see Table 1.

**Table 1:** Overview over sample sizes, completed sessions, used questionnaires and results for the key findings of previous studies

Study	Sample size	Completed sessions	Used questionnaires	Results
Edelstein et al. 2011	*n* = 132 men	One (cross-sectional design)	SOI-R	Analysis 1: No significant associations between testosterone and SOI subscales (all βs < 0.17, ps > 0.05). Analysis 2: When including interaction effects with relationship status, significant positive association between testosterone and sociosexual desire (β = 0.22, p = < 0.05) and sociosexual desire x relationship status interaction (β = 0.21, p < .05). No significant main or interaction effects of sociosexual behavior or attitudes (and relationship status; all βs < 0.19, ps > 0.05).
Gettler et al. 2019	*N* = 288 men at baseline *n* = 99 men at follow-up	*n* = 99 completed two sessions (five years apart), all other participants completed one session. Cross-sectional and longitudinal analyses (the latter with 99 observations).	SOI-R	Baseline: No significant associations between testosterone and global sociosexuality or the subscales (all *r*s < 0.10, ps > 0.100) in cross-sectional analyses. Follow-up: Small significant relationship between testosterone and global sociosexuality (r = .15, p = .01) and sociosexual attitude (r = .13, p = .03). Effects only significant for PM testosterone, not AM testosterone. No significant relationship between testosterone and sociosexual behavior (r = .10, p = .08) and desire (r = .09, p > .10) in cross-sectional analyses. Longitudinal analyses (changes in men’s T and sociosexuality scores): No significant correlations (*r*s between − 0.01 and 0.08, *p*s > 0.01).
Kordsmeyer et al. 2018	*N* = 164 men	One for relevant analyses (cross-sectional design)	SOI-R (behavior)	No significant association between testosterone and sociosexual behavior (estimate = 0.004, SE = 0.01, 95%CI = [-0.01; 0.02]
Puts et al. 2015	Study 1: *n* = 61 men Study 2: *n* = 62 men	One (cross-sectional design)	SOI-R	Positive relationship between testosterone and „sociosexual psychology“ (composite of SOI-R attitude and desire), Study 1 : β = 0.57 t = 4.08 p = .001; Study 2: β = 0.31 t = 2.27 p = .027 Negative relationship between testosterone and number of sexual partners in the last 12 months (SOI-R behavior Item 1). Study 1: β = −0.37 t = − 2.63 p = .01; Study 2: β = −0.27 t = − 2.15 p = .036
Raisanen et al. 2018	*n* = 60 men who were included in the analyses	Up to 9 sessions (but ~ 4 sessions on average and a total of 1,105 sessions). However, relevant analyses were cross-sectional.	Sexual Desire Inventory	Dyadic desire: No significant effects of testosterone (estimate = 0.41, SE = 0.73, p = .578), or cortisol (estimate = 0.33, SE = 0.60, p = .578). No interaction effect reported. Solitary desire: no significant effect of testosterone (estimate = 0.01, SE = 0.05, p = .899). Significant effect of cortisol (estimate = 0.15, SE = 0.06, p = .014), significant testosterone x cortisol interaction (estimate = -0.08, SE = 0.03, p = .011)
van Anders et al. 2007	*n* = 47 men	One (cross-sectional design)	SOI, Sexual Desire Inventory	SOI: No significant correlation with testosterone (r = .08, p = .631) Dyadic desire: No significant correlation with testosterone (r = − .05, p = .753) Solitary desire: No significant correlation with testosterone (r = .07, p = .677) Total desire: No significant correlation with testosterone (r = − .01, p = .933)
van Anders and Dunn 2009	*n* = 91 men	One (cross-sectional design)	Sexual Desire Inventory	Dyadic desire: No significant correlation with testosterone (r = .10, p = .371) Solitary desire: Significant correlation with testosterone (r = .25, p = .025) Total desire: No significant correlation with testosterone (r = .19, p = .095)
van Anders 2012	*n* = 105 men	One (cross-sectional design)	Sexual Desire Inventory	Dyadic desire: No significant correlation with testosterone (r = − .03) Solitary desire: Significant correlation with testosterone (r = − .12)

There are a variety of potential reasons for mixed results in previous studies. First, different study designs, e.g. investigating within-subjects or between-subjects effects might contribute to differences in study outcomes. Second, different analytical approaches, including different covariates, main or interaction effects, likely affect different results. Third, different studies used different questionnaires, focusing either on sexual desire or sociosexual orientation. Potentially, testosterone might be linked to some, but not to other subdimensions of sexual desire or sociosexual orientation. Fourth, besides already mentioned differences in study designs, there was a large variety in sampling, storing and analyzing hormone assays, such as freezing temperature, storage time, freeze-thaw cycles, and some studies controlled for confounding effects of diurnal changes or seasonal variability, whereas others did not.

Another important reason for inconsistencies in previous findings is that almost all studies did not investigate men’s cortisol levels, although evidence suggests that cortisol and testosterone interact and this interaction might be a better predictor for differences in social outcomes than testosterone alone (e.g. Mehta and Josephs 2010; Mehta and Prasad 2015), also known as the Dual Hormone Hypothesis. According to this hypothesis, testosterone may predict a wide range of cognition and behaviors (for an overview see Sarkar et al. 2019), but only when cortisol concentrations are low. If this is the case for sexual desire or sociosexual orientation as well, it would be crucial to investigate the interaction between testosterone and cortisol, rather than testosterone alone, as this might reveal associations that could not have been detected before. Surprisingly, only one study investigated whether the interaction between testosterone and cortisol is associated with sexual desire. This study by Raisanen and colleagues (Raisanen et al. 2018) suggests that testosterone and cortisol interact to predict men’s solitary sexual desire, but not dyadic sexual desire. Specifically, testosterone levels were positively related to solitary sexual desire, but only among men with low cortisol levels. Notably, analyses in this study were cross-sectional as well and no previous study investigated the association between a testosterone and cortisol interaction and sociosexual orientation.

As already mentioned above, considering different covariates that potentially affect hormone levels or sexual desire and sociosexual orientation, might also explain differences in previously reported results. One such potentially important variable that might affect hormone levels, as well as sexual desire or sociosexual orientation, is men’s relationship status. More precisely, one previous study reported evidence for an interaction effect of relationship status and sociosexual desire predicting testosterone levels. In this study, partnered men with higher sociosexual desire showed higher testosterone levels that were as high as testosterone levels of single men (Edelstein et al. 2011). Importantly, again, this study did not consider a potential interaction effect of testosterone and cortisol and only focused on sociosexual orientation, not on sexual desire.

In light of the mixed results for steroid hormones and both men’s sexual desire and sociosexual orientation, we investigated possible relationships between within-subject changes in men’s salivary testosterone and cortisol and their reported sexual desire (assessed using the Revised Sexual Desire Inventory, Spector et al. 1996) and sociosexual orientation (assessed using the Revised Sociosexual Orientation Inventory; Penke and Asendorpf 2008). We also investigated possible relationships between men’s average steroid hormone levels (between-subjects analyses) and their reported sexual desire and sociosexual orientation. Further, in exploratory analyses, we investigated whether men’s relationship status might have an impact on the results.

## Methods

### Participants

Sixty-one men (57 reporting being heterosexual, one reporting being homosexual, two reporting being bisexual, and one reporting being attracted to neither men nor women) participated in the study (mean age = 22.2 years, SD = 3.32 years). 31 of the participants reported to be in a committed romantic relationship, 30 reported to be single. None of these men were currently taking any form of hormonal supplements or had taken any form of hormonal supplements in the 90 days prior to participation. Participants took part in the study as part of a larger project investigating hormonal correlates of voice and face perception (Kandrik et al. 2016,2017).

### Procedure

Participants completed up to five weekly test sessions in five consecutive weeks. All participants were sampled during one semester, which reduces the influence of seasonal variability in testosterone concentrations (Stanton et al. 2011). Sessions took place between 2 pm and 5 pm to minimize diurnal variation in hormone levels (Papacosta and Nassis 2011). Fifty-seven of the participants completed more than one test session, with 50 of the participants completing all five test sessions. Unfortunately, we did not do a preplanned power simulation, but recruited as many participants as we could in a semester and went with the weekly testing sessions to match a parallel project on women (see e.g. Jones et al. 2018). Although this is not optimal, we believe that, due to our longitudinal design, we should have a better test power than most previous work. Especially for within-subjects analyses, as our number of observations (= 283) largely exceeds those of Gettler and colleagues (Gettler et al. 2019) analyses (= 99), which is the only study investigating within-subjects changes in testosterone and sociosexual orientation that has been published so far. Further, our sample size matches the one of Raisanen and colleague‘s (Raisanen et al. 2018) study (to our knowledge the only study investigating an interaction effect of testosterone and cortisol) for between-subjects analyses.

During each test session, participants provided a saliva sample via the passive drool method (Papacosta and Nassis 2011). Participants were instructed to avoid consuming alcohol and coffee in the 12 h prior to participation and to avoid eating, smoking, drinking, chewing gum, or brushing their teeth in the 60 min prior to participation. They also completed the Sexual Desire Inventory (SDI-2) and Revised Sociosexual Orientation Inventory (SOI-R) in each session. The order in which participants provided saliva samples and completed the questionnaires was fully randomized (across participants and sessions). Questionnaire responses were collected using the Experimentum interface (DeBruine 2019). For intercorrelations of this study’s main variables, see Table 2,^1^.

**Table 2:** Intercorrelations between the main study variables

	1	2	3	4	5	6	7	8	9	10	11
1 Testosterone current											
2 Cortisol current	0.26*** [0.14; 0.36]										
3 Testosterone average	− 0.00 [-0.12; 0.12]	− 0.00 [-0.12; 0.12]									
4 Cortisol average	− 0.00 [-0.12; 0.12]	− 0.00 [-0.12; 0.12]	0.54*** [0.45; 0.62]								
5 SOIR_B current	− 0.02 [-0.15; 0.10 ]	− 0.01 [-0.14; 0.11]	0.26 [0.14; 0.37]	0.16* [0.04; 0.28]							
6 SOIR_A current	0.01 [-0.11; 0.13]	0.01 [-0.11; 0.13]	0.01 [-0.11; 0.13]	− 0.14* [-0.25; -0.02]	0.41*** [0.30; 0.50]						
7 SOIR_D current	− 0.02 [-0.14; 0.10]	− 0.07 [-0.19; 0.05]	0.02 [-0.10; 0.14]	− 0.11 [-0.23; 0.00]	0.26*** [0.14; 0.37]	0.41*** [0.31; 0.50]					
8 SOIR_ful currentl	− 0.02 [-0.15; 0.10]	− 0.03 [-0.15; 0.09]	0.11 [-0.01; 0.23]	− 0.06 [-0.18; 0.06]	0.68*** [0.61; 0.74]	0.85*** [0.81; 0.88]	0.74*** [0.68; 0.79]				
9 SDI_solitary current	− 0.04 [-0.16; 0.08]	− 0.02 [-0.13; 0.11]	− 0.02 [-0.14; 0.10]	− 0.13* [-0.25; -0.01]	0.11 [-0.02; 0.23]	0.37*** [0.21; 0.43]	0.26*** [0.14; 0.37]	0.39*** [0.21; 0.43]			.
10 SDI_dyadic current	0.05 [-0.08; 0.17]	− 0.00 [-0.12; 0.12]	− 0.17** [-0.28; -0.05]	− 0.30*** [-0.41; -0.19]	0.38*** [0.27; 0.48]	0.45*** [0.35; 0.54]	0.38*** [0.28; 0.48]	0.54*** [0.44; 0.62]	0.42*** [0.32; 0.52]		
11 SDI_full current	0.01 [-0.11; 0.13]	− 0.02 [-0.14; 0.10]	− 0.16* [-0.27; 0.04]	− 0.28*** [-0.39; -0.17]	0.35*** [0.23; 0.45]	0.48*** [0.38; 0.57]	0.39*** [0.28; 0.49]	0.54*** [0.45; 0.62]	0.72*** [0.65; 0.77]	0.92*** [0.89; 0.93]	

*Note.* *p < .05, **p < .01, ***p < .001. Confidence intervals are *95%CIs*. All variables have been scaled. All correlation coefficients are pearson’s *r*. All tests were two-tailed. Testosterone current = participant’s testosterone levels from every session, Cortisol current = participant’s cortisol levels from every session, Testosterone average = averaged testosterone levels (averaged per participant), Cortisol average = averaged cortisol levels (averaged per participants), SOIR_B = SOIR behavior subscale from every session, SOIR_A = SOIR attitude subscale from every session, SOIR_D = SOIR desire subscale from every session, SOIR_full = SOIR full scale score from every session, SDI_solitary = SDI2 solitary desire subscale from every session, SDI_dyadic = SDI2 dyadic desire subscale from every session, SDI_full = SDI2 generall desire full scale score from every session. Thus, all correlation coefficient reflect the correlations between independent observations, except for the average hormone values. Importantly, these correlation coefficients ignore the nested structure of the data

### Sexual Desire Inventory (SDI-2)

The Sexual Desire Inventory (SDI-2) is a 14-item questionnaire that assesses general sexual desire (Spector et al. 1996). An example question is “When you are in romantic situations (such as a candle lit dinner, a walk on the beach, etc.), how strong is your sexual desire?”, to which participants responded using a 1 (no desire) to 9 (strong desire) scale. As well as providing a score for total sexual desire^2^ (summarizing all items, M = 60.11, SD = 16.33), the SDI-2 also provides separate scores for desire for sexual activity with another person (dyadic sexual desire by summarizing items 1 to 8, M = 38.55, SD = 10.04) and desire for sexual activity by oneself (solitary sexual desire by summarizing items 10 to 12, M = 10.63, SD = 5.11). Cronbachs alpha for the SDI-2 and it’s subscales were: dyadic sexual desire α = 0.84, solitary sexual desire α = 0.82, general sexual desire α = 0.88.

### Revised Sociosexual Orientation Inventory (SOI-R)

The Revised Sociosexual Orientation Inventory (SOI-R) is a nine-item questionnaire that assesses openness to uncommitted sexual relationships (Penke and Asendorpf 2008). Each item is answered using a 1 to 5 scale. The SOI-R has three components^2^ (desire, attitude, and behavior). The desire component consists of 3 items (e.g., “In everyday life, how often do you have spontaneous fantasies about having sex with someone you have just met?”), for which 1 on the response scale corresponds to “never” and 5 corresponds to “nearly every day” (M = 3.25, SD = 0.95). The attitude component consists of 3 items (e.g., “Sex without love is OK”), for which 1 on the response scale corresponds to “totally disagree” and 5 corresponds to “totally agree” (M = 3.52, SD = 1.15). The behavior component consists of 3 items (e.g., “With how many different partners have you had sex within the past 12 months?”), for which 1 on the response scale corresponds to “0 sexual partners” and 5 corresponds to “8 or more sexual partners” (M = 2.17, SD = 0.81). Scores for each component are calculated by averaging the individual scores for the 3 relevant items (M = 2.98, SD = 0.75). Cronbachs alpha for the SOI-R and it’s subscales were: desire α = 0.84, attitude α = 0.85, behavior α = 0.77, full scale score α = 0.83.

### Assays

Saliva samples were immediately frozen and stored at -32 °C (as recommended to us by the Salimetrics lab) for the duration of the project (~ up to max. one semester, one freeze-thaw cycle).Then, all samples were shipped frozen on dry ice to the Salimetrics Lab (Suffolk, UK) for analysis immediately after all data has been collected. There, they were kept frozen in a freezer until the day of analysis at which they were assayed using the Salivary Testosterone Enzyme Immunoassay Kit 1-2402 (M = 178.26 pg/mL, SD = 41.97 pg/mL, sensitivity < 1.0 pg/mL, intra-assay CV = 4.60%, inter-assay CV = 9.83%) and Salivary Cortisol Enzyme Immunoassay Kit 1-3002 (M = 0.19 µg/dL, SD = 0.11 µg/dL, sensitivity < 0.003 µg/dL, intra-assay CV = 3.50%, inter-assay CV = 5.08%). All assays were performed in singlets. The reported CVs were given by the Salimetrics lab. These are generic baseline CVs not specific to this dataset and thus, not calculated from the assays of our study.

Hormone levels more than three standard deviations from the sample mean or where Salimetrics indicated levels were outside the sensitivity range of the relevant ELISA were excluded from the dataset (< 1% of hormone measures were excluded for these reasons; one cortisol value and four testosterone values). The descriptive statistics given above do not include these excluded values.

For *current* hormone levels, values for each hormone were centered on their subject-specific means to isolate effects of within-subject changes in hormones. They were then standardized to achieve a distribution for each hormone from approx. − 0.5 to 0.5 to facilitate calculations in the linear mixed models. To calculate *average* hormone levels, the average value for each hormone across test sessions was calculated for each man. These values were then centered on their grand means and standardized. As a result, again the majority of the distribution for each hormone varied from − 0.5 to 0.5. Scaling within-subjects and between-subjects hormone levels is a common procedure to deal with the non-normal distribution of hormone levels. R code and resulting plots of these values are given in our Supplemental Materials (lines 164 to 197 in the R script) and show no evidence of skew.

### Analyses

We used a linear mixed model to test for possible effects of hormone levels on reported sexual desire and sociosexual orientation. Analyses were conducted using R version 3.6.1 (R Core Team 2016), with lme4 version 1.1–21 (Bates et al. 2014) and lmerTest version 3.1-0 (Kuznetsova et al. 2015). Standardized effect size estimates were computed using sjPlot version 2.8.3 (Lüdecke 2018). Data files and analysis scripts are publicly available at https://osf.io/d79px/.

## Results

Scores on the SDI-2 subscales and each subscale of the SOI-R were analyzed separately. Predictors were current testosterone, current cortisol, and their interaction, and average testosterone, average cortisol, and their interaction. Importantly, results on current hormone levels reflect within-subjects changes in hormones over time (longitudinal analyses), whereas results on average hormone levels reflect between-subjects differences (cross-sectional analyses). Results do not change when computing within- and between-subjects effects in separate models. No covariates were included in the model. Random slopes were specified maximally following Barr et al. (2013) and Barr (2013). Some models yield convergence warnings that were rather low above the threshold. However, when reducing these models (adding main effect slopes instead of interaction effect slopes), these warnings disappeared, but results were robust (and virtually identical). Thus, we decided to stay with the maximum specification. Full model specifications and full results for each analysis are given in our Supplemental Information.

### Sexual Desire (SDI-2)

There were no significant effects of current hormone levels or average hormone levels on total sexual desire (total score on the SDI-2), solitary sexual desire (score of the solitary subscale on the SDI-2) or dyadic sexual desire (score of the dyadic subscale of the SDI-2), see Table 3.

**Table 3:** Multilevel regression analyses of sexual desire (total sexual desire, solitary sexual desire or dyadic sexual desire) as a function of within-and between-men testosterone, cortisol and their interactions

	estimate	SE	95% CI	Std. β	Std. 95% CI	t	*p*
Total sexual desire							
Current testosterone	1.29	2.79	[-4.24; 7.15]	0.01	[-0.03; 0.05]	0.46	0.648
Current cortisol	-2.07	2.24	[-6.70; 2.74]	-0.02	[-0.06; 0.02]	-0.93	0.375
Current testosterone x current cortisol	27.04	17.94	[-8.50; 63.06]	0.03	[-0.01; 0.07]	1.51	0.137
Average testosterone	2.09	11.81	[-21.43; 25.61]	0.02	[-0.26; 0.30]	0.18	0.860
Average cortisol	-17.12	14.74	[-46.44; 12.29]	-0.16	[-0.43; 0.11]	-1.16	0.250
Average testosterone x average cortisol	-74.81	52.57	[-179.37; 29.84]	-0.14	[-0.33; 0.05]	-1.42	0.160
Solitary sexual desire							
Current testosterone	-1.65	0.93	[-3.51; 0.21]	-0.04	[-0.08; 0.00]	-1.78	0.077
Current cortisol	0.41	0.77	[-1.13; 1.95]	0.01	[-0.03; 0.06]	0.53	0.597
Current testosterone x current cortisol	2.25	6.27	[-10.29; 14.79]	0.01	[-0.04; 0.05]	0.36	0.720
Average testosterone	3.69	3.55	[-3.41; 10.79]	0.14	[-0.13; 0.42]	1.04	0.303
Average cortisol	-4.06	4.44	[-12.94; 4.82]	-0.12	[-0.38; 0.14]	-0.91	0.365
Average testosterone x average cortisol	-28.45	15.80	[-60.05; 3.15]	-0.17	[-0.36; 0.02]	-1.80	0.077
Dyadic sexual desire							
Current testosterone	3.96	2.35	[-0.74; 8.66]	0.05	[-0.01; 0.10]	1.69	0.102
Current cortisol	-1.18	1.67	[-4.52; 2.16]	-0.02	[-0.07; 0.03]	-0.71	0.481
Current testosterone x current cortisol	22.27	13.63	[-4.99; 49.53]	0.04	[-0.01; 0.09]	1.63	0.104
Average testosterone	-0.75	7.27	[-15.29; 13.79]	-0.02	[-0.30; 0.26]	-0.10	0.918
Average cortisol	-10.17	9.08	[-28.33; 7.99]	-0.15	[-0.42; 0.12]	-1.12	0.267
Average testosterone x average cortisol	-38.65	32.35	[-103.35; 26.05]	-0.12	[-0.31; 0.07]	-1.20	0.237

*Note*. Current hormone levels reflect within-men hormone effects, average hormone levels reflect between-men hormone effects. Estimates are unstandardized effect size estimates

### Sociosexual Orientation (SOI-R)

**Fig. 1:**
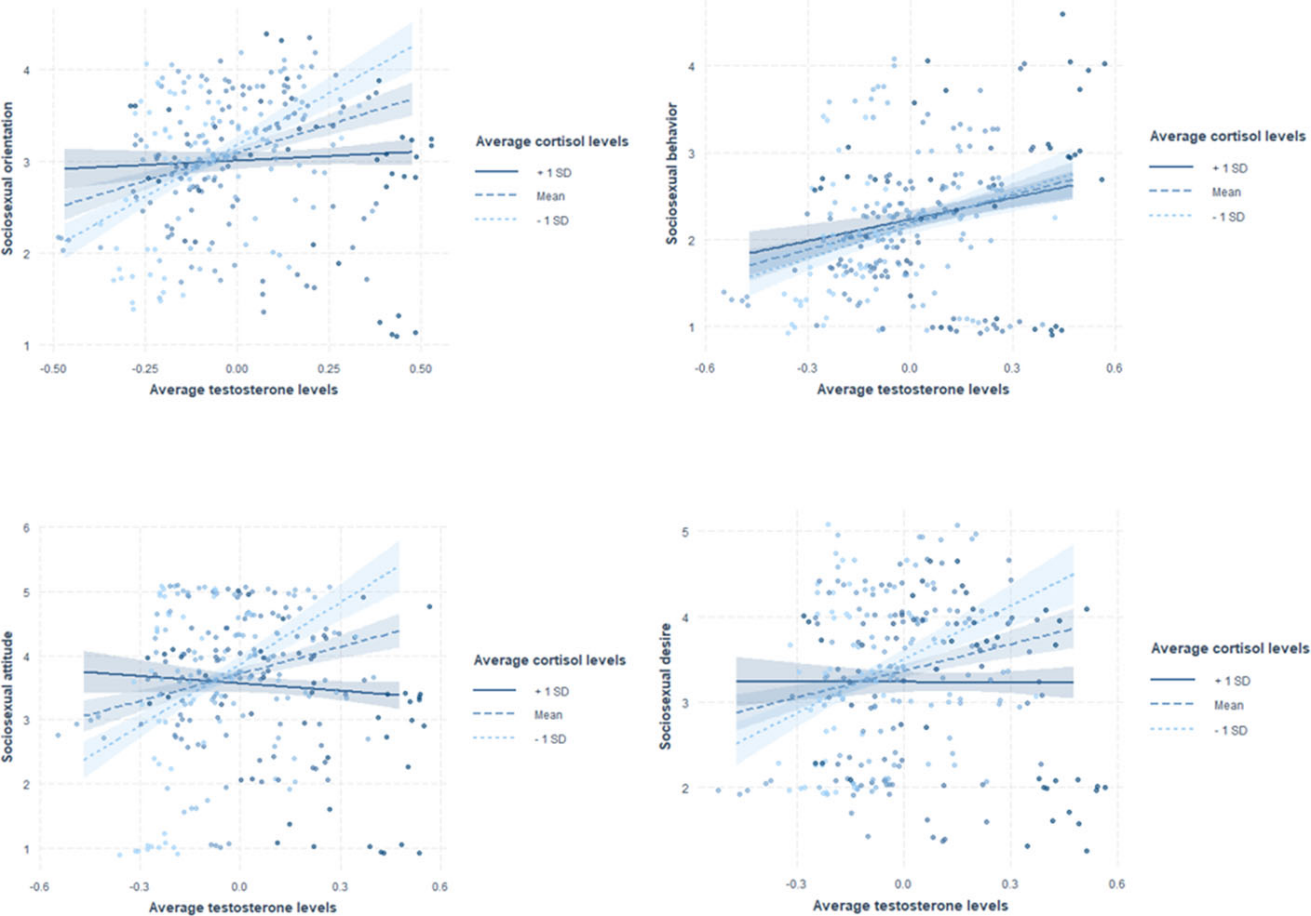
Interaction effects between average testosterone and average cortisol levels on the SOI-R full scale score and facets. Note: Hormone levels are scaled. Average cortisol levels are centered for the purpose of illustration. Shades show the 95% CI. X-axis and y-axis are truncated

There were no significant effects of current hormone levels or main effects of average hormone levels on sociosexual orientation (total score on the SOI-R) or the three subfacets behavior, attitude and desire, besides a significant negative effect of current cortisol on sociosexual desire, suggesting that men with lower current cortisol levels had higher sociosexual desire (Table 4). However, this effect has to be interpreted with caution as the upper bound of the 95%CI is very close to zero and the standardized effect size estimate is very small. Further, there were significant negative interactions between average testosterone and average cortisol levels on sociosexual orientation, attitude and desire, but no significant interaction effect on sociosexual behavior. Only men with relatively low levels of cortisol showed a positive relationship between testosterone and sociosexual orientation, attitude and desire (see Fig. 1).

**Table 4:** Multilevel regression analyses of sociosexuality (sociosexual orientation, sociosexual behavior, sociosexual attitude or sociosexual desire) as a function of within-and between-men testosterone, cortisol and their interactions

	estimate	SE	95% CI	Std. β	Std. 95% CI	t	*p*
Sociosexual orientation							
Current testosterone	-0.05	0.13	[-0.31; 0.21]	-0.01	[-0.05; 0.03]	-0.38	0.703
Current cortisol	-0.13	0.12	[-0.37; 0.11]	-0.03	[-0.08; 0.02]	-1.06	0.297
Current testosterone x current cortisol	0.47	0.87	[-1.27; 2.21]	0.02	[-0.03; 0.06]	0.54	0.589
Average testosterone	0.91	0.51	[-0.11; 1.93]	0.25	[-0.02; 0.52]	1.79	0.079
Average cortisol	-0.20	0.64	[-1.48; 1.08]	-0.03	[-0.28; 0.23]	-0.31	0.755
Average testosterone x average cortisol	-6.17	2.27	[-10.71; -1.63]	-0.25	[-0.43; -0.07]	-2.72	0.009
Sociosexual behavior							
Current testosterone	-0.14	0.14	[-0.42; 0.14]	-0.02	[-0.06; 0.02]	-1.00	0.318
Current cortisol	-0.04	0.12	[-0.28; 0.20]	-0.01	[-0.05; 0.04]	-0.37	0.715
Current testosterone x current cortisol	1.70	1.00	[-0.30; 3.70]	0.04	[-0.01; 0.09]	1.71	0.109
Average testosterone	0.76	0.61	[-0.46; 1.98]	0.19	[-0.11; 0.50]	1.24	0.220
Average cortisol	0.29	0.77	[-1.27; 1.83]	0.06	[-0.23; 0.34]	0.38	0.705
Average testosterone x average cortisol	-0.32	2.72	[-5.76; 5.12]	-0.01	[-0.22; 0.19]	-0.12	0.906
Sociosexual attitude							
Current testosterone	0.12	0.26	[-0.40; 0.64]	0.01	[-0.04; 0.07]	0.45	0.654
Current cortisol	0.09	0.22	[-0.35; 0.53]	0.01	[-0.04; 0.07]	0.44	0.666
Current testosterone x current cortisol	-1.50	1.54	[-4.58; 1.58]	-0.03	[-0.08; 0.03]	-0.98	0.329
Average testosterone	1.24	0.74	[-0.24; 2.72]	0.21	[-0.04; 0.46]	1.67	0.100
Average cortisol	-0.29	0.93	[-2.15; 1.57]	-0.03	[-0.27; 0.21]	-0.32	0.753
Average testosterone x average cortisol	-11.83	3.30	[-18.43; 5.23]	-0.31	[-0.48; -0.14]	-3.59	0.001
Sociosexual desire							
Current testosterone	-0.00	0.21	[-0.42; 0.42]	-0.00	[-0.05; 0.05]	-0.01	0.985
Current cortisol	-0.49	0.24	[-0.97; -0.01]	-0.08	[-0.16; -0.00]	-2.07	0.044
Current testosterone x current cortisol	1.83	1.53	[-1.23; 4.89]	0.04	[-0.03; 0.10]	1.08	0.294
Average testosterone	0.91	0.63	[-0.35; 2.17]	0.19	[-0.07; 0.45]	1.45	0.153
Average cortisol	-0.75	0.79	[-2.33; 0.83]	-0.11	[-0.36; 0.14]	-0.95	0.348
Average testosterone x average cortisol	-6.36	2.81	[-11.98; -0.74]	-0.20	[-0.38; -0.03]	-2.27	0.027

*Note*. Current hormone levels reflect within-men hormone effects, average hormone levels reflect between-men hormone effects. Estimates are unstandardized effect size estimates

### Robustness Checks

Repeating the analyses described above, this time removing men who did not report being heterosexual from the dataset, showed the same patterns of significant and non-significant results.

### Exploratory Analyses

We conducted additional exploratory analyses that match analyses from previous studies. First, we did the exact same cross-sectional analyses as in Puts and colleagues (Puts et al. 2015), and additionally replicated these analyses with our longitudinal data. For this purpose, we first computed a multiple regression with testosterone levels as outcome and a composite score of sociosexual attitude and desire, as well as number of sexual partners in the last 12 months (sociosexual behavior Item 1) as predictors, only using the data from the first session per participant. Analyses revealed no significant effects of the sociosexual attitude and desire composite score (estimate = -0.02, SE = 0.02, t = -0.93, p = .356, 95%CI = [-0.07; 0.03], std. β = -0.13, std. 95%CI = [-0.42; -0.15]), or number of sexual partners in the last 12 months (estimate = -0.02, SE = 0.02, t = -0.99, p = .329, 95%CI = [-0.07; 0.02], std. β = -0.14, std. 95%CI = [-0.43; -0.15]). Effect size estimates were rather small. Next, we repeated the model from Puts and colleagues (Puts et al. 2015) using our longitudinal data in a multilevel model with maximum random slope specification. Again, analyses revealed no significant effects with small estimates and confidence intervals narrow around zero. More precisely, there was no significant effect of sociosexual attitude and desire composite score (estimate = -0.00, SE = 0.01, t = -0.17, p = .866, 95%CI = [-0.02; 0.02], std. β = -0.01, std. 95%CI = [-0.14; 0.12]), or number of sexual partners in the last 12 months (estimate = -0.00, SE = 0.01, t = -0.21, p = .836, 95%CI = [-0.02; 0.02], std. β = -0.01, std. 95%CI = [-0.14; 0.11]) on current testosterone levels.

Second, we did the same analyses as in Edelstein and colleagues (Edelstein et al. 2011), investigating associations between testosterone levels and the interaction between relationship status and the three facets of the SOI-R. Again, we computed a cross-sectional analysis first. For this purpose, we conducted a multiple regression with average testosterone level per participant as outcome and the three facets desire, behavior and attitude, each interacting with relationship status, as predictors. Following Edelstein and colleagues (Edelstein et al. 2011), relationship status was contrast coded (-1 = singles, 1 = partnered). Results are displayed in Table 5. There were no significant main effects of sociosexual desire, sociosexual behavior, and sociosexual attitude, but a significant negative main effect of relationship status, suggesting that single men had higher levels of testosterone. This finding is in line with the results by Edelstein and colleagues (Edelstein et al. 2011) who also report higher testosterone levels in single men, compared to partnered men. Further, there were no significant interaction effects of relationship status and any of the three SOI-R facets. The non-significant interaction effects for sociosexual behavior and attitudes are also in line with Edelstein and colleagues (Edelstein et al. 2011) results, whereas our non-significant effect contrasts the previous results.

**Table 5:** Multiple (cross-sectional) and multilevel (longitudinal) regression analyses of testosterone levels as a function of sociosexual behavior, attitude, desire, relationship status and their interactions, following Edelstein et al. (2011)

	estimate	SE	95% CI	Std. β	Std. 95% CI	t	*p*
Cross-sectional model							
SOI-R desire	0.00	0.04	[-0.09; 0.09]	0.00	[-0.41; 0.41]	0.01	0.995
SOI-R behavior	0.05	0.07	[-0.08; 0.18]	0.23	[-0.33; 0.80]	0.84	0.407
SOI-R attitude	-0.03	0.04	[-0.12; 0.05]	-0.19	[-0.66; 0.27]	-0.84	0.407
Relationship status	-0.48	0.22	[-0.92; -0.04]	-0.53	[-1.08; -0.02]	-2.18	0.034
SOI-R desire x relationship status	0.03	0.06	[-0.10; 0.16]	0.14	[-0.47; 0.74]	0.45	0.654
SOI-R behavior x relationship status	0.05	0.08	[-0.10; 0.21]	0.23	[-0.44; 0.89]	0.68	0.500
SOI-R attitude x relationship status	0.05	0.06	[-0.07; 0.16]	0.27	[-0.37; 0.90]	0.85	0.402
Longitudinal model							
SOI-R desire	-0.00	0.01	[-0.02; 0.02]	-0.03	[-0.23; 0.16]	-0.32	0.748
SOI-R behavior	0.01	0.02	[-0.03; 0.05]	0.04	[-0.21; 0.28]	0.30	0.766
SOI-R attitude	-0.01	0.01	[-0.03; 0.01]	-0.05	[-0.28; 0.17]	-0.47	0.640
Relationship status	-0.02	0.07	[-0.16; 0.12]	-0.03	[-0.28; 0.23]	-0.27	0.790
SOI-R desire x relationship status	-0.03	0.02	[-0.07; 0.01]	-0.02	[-0.30; 0.26]	-0.14	0.890
SOI-R behavior x relationship status	-0.01	0.02	[-0.05; 0.03]	-0.09	[-0.39; 0.21]	-0.58	0.561
SOI-R attitude x relationship status	0.02	0.02	[-0.02; 0.06]	0.14	[-0.16; 0.44]	0.91	0.365

*Note*. Estimates are unstandardized effect size estimates

Next, we repeated the model from Edelstein and colleagues (2011) using our longitudinal data in a multilevel model with maximum random slope specification and current testosterone levels as outcomes. There were no significant main effects of sociosexual desire, behavior, attitude, or relationship status. Further, there were no significant interaction effects of relationship status and any of the three SOI-R facets (Table 5).

Third, as the results described above indicate that relationship status may influence between-subjects hormone levels, and as suggested in the review process, we repeated all of our main models adding relationship status as a covariate. Three models revealed a positive main effect of relationship status with medium to large effect sizes, in that men in relationships reported generally higher dyadic sexual desire (estimate = 7.32, SE = 2.43, t = 3.01, p = .004, 95%CI = [2.46; 12.18], std. β = 0.73, std. 95%CI = [0.25; 1.20]), a higher sexual desire full scale score (estimate = 11.61, SE = 3.97, t = 2.92, p = .005, 95%CI = [3.67; 19.55], std. β = 0.71, std. 95%CI = [0.23; 1.18]) and higher sociosexual behavior (estimate = 0.49, SE = 0.21, t = 2.31, p = .025, 95%CI = [0.07; 0.91], std. β = 0.62, std. 95%CI = [0.09; 1.14]). The main effect of relationship status was non-significant in all other models (*p*s between 0.10 and 0.68). Moreover, two models revealed a positive main effect of average testosterone, in that men with higher testosterone levels on average reported higher sociosexual behavior (estimate = 1.24, SE = 0.60, t = 2.07, p = .043, 95%CI = [0.04; 2.44], std. β = 0.32, std. 95%CI = [0.02; 0.62]) and a higher sociosexuality full scale score (estimate = 1.12, SE = 0.52, t = 2.15, p = .036, 95%CI = [0.08; 2.16], std. β = 0.31, std. 95%CI = [0.03; 0.58]). Effect size estimates suggest small effects. However, these effects would not be robust when controlling for multiple testing. All other results were virtually identical to those reported in the main analyses, details can be found in the supplementary material.

## Discussion

Our longitudinal analyses showed no clear associations of within-subject changes in men’s testosterone, cortisol, or their interaction on any aspects of sociosexuality or sexual desire. There was a weak negative effect of current cortisol on sociosexual desire, but this was not robust to correction for multiple testing and the upper bound of the confidence interval was very close to zero (uncorrected p-value = 0.044; std. 95%CI = [-0.16; -0.00]). These results are in line to Gettler and colleagues (Gettler et al. 2019) findings that changes in men’s testosterone levels are not related to changes in sociosexuality scores. Further, they suggest no compelling evidence that changes in men’s cortisol levels or the interaction between testosterone and cortisol are linked to changes in sociosexuality or sexual desire, an association which, to our knowledge, has not been investigated in any previous study.

Our between-subjects analyses of responses on the SDI-2 also showed no compelling evidence for cross-sectional associations between aspects of men’s sexual desire and average steroid hormones. Thus, our results are in contrast to previous findings in which men with higher average testosterone reported greater solitary sexual desire (van Anders and Dunn 2009). While our effect size estimate was in the same positive direction as the effect reported by van Anders and Dunn (2009), the standardized effect size estimate was very small (β = 0.14), and even smaller than the one reported by van Anders and Dunn (2009) (r = .25). Interestingly, in a different study, van Anders (2012) reports a significant correlation of between-subjects solitary sexual desire and testosterone levels, that is in the opposite direction (r = − .12) as the one reported by van Anders and Dunn (2009). Two other studies (Raisanen et al. 2018; van Anders et al. 2007) report no significant associations of testosterone and solitary sexual desire. Overall, the pattern of results rather suggests that this effect might not be robust.

However, our results for cross-sectional associations between aspects of men’s dyadic sexual desire or overall sexual desire and steroid hormones are consistent with similar results that have been reported in other studies (Raisanen et al. 2018; van Anders et al. 2007; van Anders and Dunn 2009; van Anders 2012). Collectively, these results suggest that associations between average steroid hormone levels and sexual desire in men may not be robust, or that studies might have been underpowered to detect small significant associations between testosterone and solitary sexual desire.

Some previous studies have reported that men with higher average testosterone levels score higher on sociosexual desire (Edelstein et al. 2011; Puts et al. 2015), and lower on sociosexual behavior (Puts et al. 2015), but only when including their relationship status or controlling for other facets of sociosexuality. These results have been interpreted as evidence for a feedback loop in which rising testosterone levels increase sociosexual desire, but that engaging in sexual behavior causes men’s testosterone levels to fall (Puts et al. 2015). Neither our longitudinal nor cross-sectional analyses of men’s sociosexuality support this proposal. When repeating Puts and colleagues (Puts et al. 2015) analyses with our data (with a comparable sample size for cross-sectional analyses), the resulting effect size estimates were very close to or even estimated as being zero. We did not find compelling evidence that men’s average testosterone levels and sociosexuality are associated, which is consistent with similar results reported in other studies (Kordsmeyer et al. 2018; van Anders et al. 2007).

Intriguingly, we found that average testosterone was positively related to sociosexual attitudes, sociosexual desires, and global sociosexual orientation (i.e., total scores on the SOI-R) among men with relatively low cortisol. Although we did not predict this result, we note here that the interactions between average testosterone and average cortisol for sociosexual attitudes and global sociosexual orientation would be significant even if Bonferroni-corrected for multiple comparisons. The interaction between average testosterone and average cortisol levels was non-significantly associated with solitary desire, dyadic desire and overall sexual desire in our study. To our knowledge, only one previous study investigated between-men associations of dyadic desire or solitary desire and a testosterone x cortisol interaction (Raisanen et al. 2018). Whereas this study does not report an interaction effect of average testosterone x average cortisol and dyadic desire, it reports a very small, but significant association between negative average testosterone x average cortisol and solitary desire (estimate = -0.08, p = .011). The interaction effect we found was non-significant, but it was in the same direction as the one reported by Raisanen and colleagues (Raisanen et al. 2018) with a p-value slightly above the significance threshold and a rather small effect size estimate (std. β = -0.17, std. 95%CI = [-0.36; 0.02], p = .078). Thus, although our sample size (N = 61) was comparable to the sample size (N = 60) of Raisanen and colleagues (Raisanen et al. 2018), it is possible that our non-significant result is a false negative due to insufficient test power.

To sum this up, the overall pattern of results in our (and the previous study) suggests that the interaction of average testosterone and average cortisol is related to differences in at least some aspects of sociosexual orientation and sexual desire. Some previous research suggests that the combination of high testosterone and low cortisol is associated with status-related behaviors (see Mehta and Prasad 2015, for a review of this Dual Hormone Hypothesis). If this is the case, our results present preliminary evidence that attitudes to uncommitted sexual relationships might be similarly related to high testosterone and low cortisol. Further research would be necessary to shed further light on this possibility. Previous studies investigating possible associations between steroid hormones and men’s sociosexuality may not have detected these relationships because most studies did not assess cortisol at all (see Table 1), or they did not consider the interaction between average testosterone and average cortisol (Kordsmeyer et al. 2018; van Anders et al. 2007).

Our results may have some clinical implications. While the overall pattern of whether the treatment of testosterone leads to an increase of men’s libido is mixed (Petering and Brooks 2017), we did not find compelling evidence for a link between testosterone levels and sexual desire or sociosexual orientation in healthy men. Whereas our results cannot answer the question whether testosterone prescriptions lead to clinically significant benefits for men with sexual dysfunction, they do challenge the underlying assumption that natural changes in testosterone are related to an increase in sexual desire or sociosexual orientation. While we only detected between-men interaction effects of testosterone and cortisol, we still think that it might be interesting for future clinical studies to consider that testosterone and cortisol might be mutually related to men’s libido. A prescription of hormones does lead to larger changes in hormone levels than naturally occurring variation in the current study (although we already interpret the natural variation of sometimes more than 2 SDs within-participant as meaningful), thus possibly also leading to within-subjects effects. As a consequence, it should be investigated whether actively trying to reduce or suppress cortisol levels, while raising testosterone levels, might increase men’s libido, based on assumptions of the Dual Hormone Hypothesis.

While overall being in line with the results of most previous studies, we would like to mention more potential reasons why some of our results might differ from those of previous studies and give some directions for future research. Besides the fact that samples and analyses differ in various characteristics (e.g. sample sizes, cultural differences, longitudinal vs. cross-sectional analyses), there might have been important differences in used research methods, especially regarding hormonal assays. Importantly, a recent study reported that differences in storage time or freeze-thaw cycles might already have a high impact on hormone assay results (Prasad et al. 2019). Further, if sampling takes part over several months or sampling sessions differ seasonally, seasonal variability in testosterone levels might be another explanation for differences in results (Stanton et al. 2011). Most of the previous studies did not provide such detailed information on their sampling specifity, which makes it hard to evaluate differences between their and our sampling procedure. However, probably most importantly, more and more research reports that analysis methods of hormone assays may have a large impact on results (Prasad et al. 2019; Schultheiss et al. 2019; Welker et al. 2016). More precisely, analyzing hormone samples via mass spectrometry (*LCMS*) is seen as the gold standard, but all studies so far, including ours, used recently criticized immunoassays (for a detailed overview see Schultheiss et al. 2019). Moreover, most studies even used different types of immunoassays as used in the current study (Edelstein et al. 2011; Kordsmeyer et al. 2018; Raisanen et al. 2018; van Anders 2012; van Anders and Dunn 2009; van Anders et al. 2007). This might probably not only explain differences in specific results, but may also raise general uncertainty in interpreting results (e.g. as Salimetrics and IBL kits often overestimate salivary testosterone concentrations; Welker et al. 2016 and a lack of validity contributes to measurement variance; Schultheiss et al. 2019) and suggests that a focus on reliable methods is crucial for future research.

Strengths of the current study include the longitudinal analyses and consideration of testosterone, cortisol, and their interaction. However, there are limitations that could be addressed in future work. First, replicating the cross-sectional findings for sociosexuality and sexual desire in a larger sample may clarify whether our results are robust or false positives or false negatives. While most previous studies had sample sizes in a comparable range as ours, all samples might have been too small to have a high enough statistical test power to detect very small effects or higher order interactions. Therefore, we suggest that future studies should do a preplanned power simulation according to the expected effect size and, if possible, even preregister their design and analyses alongside their estimated test power. Second, our methods for analysing hormone levels might not have been optimal. Using immunoassays might have led to an overestimation of salivary testosterone levels (Schultheiss et al. 2019) and unreliable hormone assays might be one reason for differences in results. Thus, future studies should aim for the gold standard method LCMS to analyse their hormone samples. We also suggest that hormone analyses should be done in duplicates rather than in singlets and (blind) low and high control samples should be added to the analyses to obtain reliable intra-assay and inter-assay CVs. In contrast, the CVs we reported here were not based on our data, as analyses were done in singlets and we did not add control samples to the assays, which limits the interpretability of our results. Additionally, using a storage temperature of -80 °C would have been ideal, whereas we only used a storage temperature of -32 °C, which has previously been reported to induce error variance for salivary testosterone levels estimated by immunoassays (Granger et al. 2004). Further, to increase replicability and transparency, future studies should emphasize to report more detailed information on their hormone assay methods and consider recently suggested guidelines in assessing hormones, such as few freeze-thaw cycles, short storage times and potentially controlling for seasonal variation (Prasad et al. 2019; Stanton et al. 2011). Third, we did not report any meta-analytic results including all previous studies and equivalence tests to test potential null results, as we think this is beyond the scope of our paper. However, to get a more precise picture of the current evidence on the association of (socio-) sexual desire, testosterone and cortisol, we recommend that future studies should report meta-analytic results and, for null results, equivalence tests with previously defining a smallest effect size of interest (Lakens et al. 2018).

In conclusion, we did not find compelling evidence that aspects of men’s sexual desire are linked to their steroid hormones. However, we did find evidence that aspects of men’s sociosexual orientation, most notably their attitude to casual sex, was predicted by the interaction between average testosterone and average cortisol. Men with higher average testosterone levels reported more positive attitudes to casual sex, but only if they also had relatively low average cortisol. While such a pattern of results is arguably consistent with the Dual Hormone Hypothesis of men’s competitive behaviors, further work is needed to establish whether this pattern of results is robust.

## Electronic Supplementary Material

ESM 1 (PDF 179 KB)

## Footnotes

1 Please note that these correlation coefficients do not account for the nested structure of the data. Thus, they should be cautiously interpreted and cannot be seen as equivalent to the results from the multilevel models as reported in the results part.

2 Reported means and standard deviations are across all participants and sessions.
