# Supplementary material for: Are Sexual Desire and Sociosexual Orientation Related to Men’s Salivary Steroid Hormones?
Source: Adapt Human Behav Physiol. 2020 Aug 24;6(4):447–66. doi: 10.1007/s40750-020-00148-y (PMC7553893; doi:10.1007/s40750-020-00148-y)
Supplement: Supplementary file 1 — (PDF 179 KB) [file 40750_2020_148_MOESM1_ESM.pdf]

testosterone and sexual desire in men

Julia Stern

12 June 2020

```
library(dplyr)
```

```
##
## Attaching package: 'dplyr'
```

```
## The following objects are masked from 'package:stats':
##
##     filter, lag
```

```
## The following objects are masked from 'package:base':
##
##     intersect, setdiff, setequal, union
```

```
library(tidyverse)
```

```
## -- Attaching packages ---------------------------------------------------------
- tidyverse 1.2.1 --
```

```
## v ggplot2 3.2.1     v readr   1.3.1
## v tibble  2.1.3     v purrr   0.3.2
## v tidyr   1.0.0     v stringr 1.4.0
## v ggplot2 3.2.1     v forcats 0.4.0
```

```
## -- Conflicts ------------------------------------------------------------- tidy
verse_conflicts() --
## x dplyr::filter() masks stats::filter()
## x dplyr::lag()    masks stats::lag()
```

```
library(lmerTest)
```

```
## Loading required package: lme4
```

```
## Loading required package: Matrix
```

```
##
## Attaching package: 'Matrix'
```

```
## The following objects are masked from 'package:tidyr':
##
##     expand, pack, unpack
```

```
##
## Attaching package: 'lmerTest'
```

```
## The following object is masked from 'package:lme4':
##
##     lmer
```

```
## The following object is masked from 'package:stats':
##
##     step
```

```
library(psych)
```

```
##
## Attaching package: 'psych'
```

```
## The following objects are masked from 'package:ggplot2':
##
##     %+%, alpha
```

```
library(ggplot2)
library(reshape2)
```

```
## Warning: package 'reshape2' was built under R version 3.6.3
```

```
##
## Attaching package: 'reshape2'
```

```
## The following object is masked from 'package:tidyr':
##
##     smiths
```

```
library(sjPlot)
```

```
## Warning: package 'sjPlot' was built under R version 3.6.3
```

```
## Learn more about sjPlot with 'browseVignettes("sjPlot")'.
```

```
library(sjmisc)
```

```
## Learn more about sjmisc with 'browseVignettes("sjmisc")'.
```

```
##
## Attaching package: 'sjmisc'
```

```
## The following object is masked from 'package:purrr':
##
##     is_empty
```

```
## The following object is masked from 'package:tidyr':
##
##     replace_na
```

```
## The following object is masked from 'package:tibble':
##
##     add_case
```

Descriptive statistics and full output for all analyses. This document also includes analyses of reported anxiety levels that are not reported in the main text.

```
# Load Data
setwd("C:/Users/jjuenge/Desktop/data")
data <- read.csv2("C:/Users/jjuenge/Desktop/data/testosterone_cortisol_desire_relat
ionship.csv")


###numbers as numeric, categories as factor variables
for(i in c(3,8:10,12:36)){
  data[,i] <- as.numeric(as.character(data[,i]))
}

for(i in c(1,2)){
  data[,i] <- as.factor(data[,i])
}

#exclude participants who reported using hormonal supplements (n = 7)

data <- data[- grep("1001", data$hm_id),]
data <- data[- grep("1006", data$hm_id),]
data <- data[- grep("1023", data$hm_id),]
data <- data[- grep("1026", data$hm_id),]
data <- data[- grep("1029", data$hm_id),]
data <- data[- grep("1031", data$hm_id),]
data <- data[- grep("1054", data$hm_id),]
```

Basic Descriptive Information for Sample

The number of sessions completed per man

```
data %>%
  group_by(hm_id) %>%
  summarise(
    sessions = n_distinct(date)
  ) %>%
  group_by(sessions) %>%
  summarise(
    n = n()
  )
```

```
## # A tibble: 5 x 2
##   sessions     n
##      <int> <int>
## 1        1     4
## 2        2     2
## 3        3     2
## 4        4     3
## 5        5    50
```

Mean age for the sample

```
data %>%
  group_by(hm_id) %>%
  summarise(age = mean(age, na.rm = T)) %>%
  ungroup() %>%
  group_by() %>%
  summarise(
      n = n(),
      mean_age = mean(age, na.rm = TRUE),
      sd_age =   sd(age, na.rm = TRUE),
      se_age =   se(age, na.rm = TRUE)
  ) %>%
  mutate_all(round, 2)
```

```
## # A tibble: 1 x 4
##       n mean_age sd_age se_age
##   <dbl>    <dbl>  <dbl>  <dbl>
## 1    61     22.2   3.32   0.43
```

Data Processing

Exclude hormone outliers

```
# calculate means and SDs
testosterone_mean <- mean(data$testosterone)
testosterone_sd <- sd(data$testosterone)
cortisol_mean <- mean(data$cortisol)
cortisol_sd <- sd(data$cortisol)

# set values > 3SD from the mean to NA
data_final <- data %>%
  mutate (
    testosterone = ifelse (testosterone > testosterone_mean + 3*testosterone_sd |
                  testosterone < testosterone_mean - 3*testosterone_sd, NA, testost
erone),
    cortisol = ifelse (cortisol > cortisol_mean + 3*cortisol_sd |
                  cortisol < cortisol_mean - 3*cortisol_sd, NA, cortisol)
  )

# determine how many values were excluded
data_final %>%
  group_by(hm_id, date) %>%
  summarise(
    t = is.na(mean(testosterone)),
    c = is.na(mean(cortisol))
  ) %>%
  ungroup() %>%
  select(t:c) %>%
  gather('hormone','na', t:c) %>%
  group_by(hormone) %>%
  summarise(
    'valid' = n() - sum(na),
    'excluded' = sum(na)
  ) %>%
  arrange(hormone)
```

```
## # A tibble: 2 x 3
##   hormone valid excluded
##   <chr>   <int>    <int>
## 1 c         275        1
## 2 t         272        4
```

```
# Calculate average hormones for each participant
data_avg <- data_final %>%
  group_by(hm_id) %>%
  summarise(
    avg_testosterone = mean(testosterone, na.rm = TRUE),
    avg_cortisol = mean(cortisol, na.rm = TRUE)
  ) %>%
  group_by() %>%
  # divide by constants to make range approx -.5 to +.5 for lmer
  mutate(
    avg_testosterone.s = (avg_testosterone - mean(avg_testosterone, na.rm=TRUE)) /
180,
    avg_cortisol.s = (avg_cortisol-mean(avg_cortisol, na.rm=TRUE)) / 0.5
  )
```

Centre and scale hormones

Centre hormones on subject-specific means, and bring values between -0.5 and 0.5 to facilitate calculations in linear mixed effects models. This graph illustrates that testosteroneosterone and cortisolisol values are not skewed.

```
# centre hormones within-subject
# divide by same constants above to make range approx -.5 to +.5 for lmer
data_scaled <- data_final %>%
  group_by(hm_id) %>%
  mutate(
    testosterone.s = (testosterone-mean(testosterone, na.rm=TRUE)) / 180,
    cortisol.s = (cortisol-mean(cortisol, na.rm=TRUE)) / 0.5
  ) %>%
  ungroup() %>%
  left_join(data_avg, by="hm_id")
```

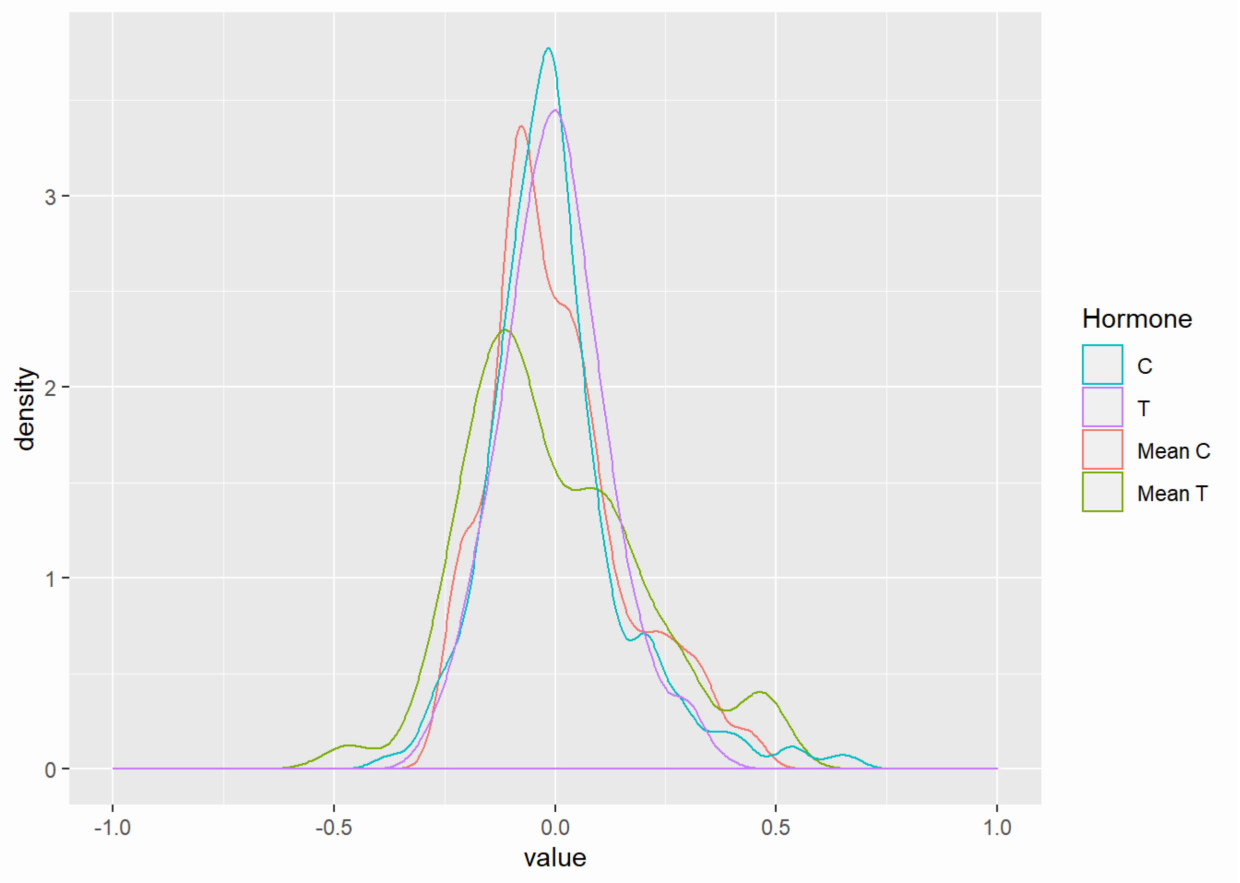

Mean hormone levels

```
data_scaled %>%
  group_by(hm_id, date, age, testosterone, cortisol) %>%
  summarise(n = n()) %>%
  ungroup() %>%
  group_by() %>%
  summarise(
      mean_testosterone = mean(testosterone, na.rm = TRUE),
      sd_testosterone =   sd(testosterone, na.rm = TRUE),
      se_testosterone =   se(testosterone, na.rm = TRUE),
      mean_cortisol = mean(cortisol, na.rm = TRUE),
      sd_cortisol =   sd(cortisol, na.rm = TRUE),
      se_cortisol =   se(cortisol, na.rm = TRUE)
  ) %>% gather("stat", "value", 1:length(.)) %>%
    mutate(value = round(value, 4)) %>%
    separate(stat, c("stat", "hormone")) %>%
    spread(stat, value)
```

```
## # A tibble: 2 x 4
##   hormone        mean     sd     se
##   <chr>         <dbl>  <dbl>  <dbl>
## 1 cortisol      0.190  0.107 0.0064
## 2 testosterone 178.    41.4   2.50
```

Sexual desire Analyses

Notes:

SOI already reverse scored.

Average items to get subscale values for each facet

Then average all items for the SOI-R full scale score

```
data_scaled$SOIR_behavior <- rowMeans(data_scaled[,c(12:14)])
data_scaled$SOIR_attitude <- rowMeans(data_scaled[,c(15:17)])
data_scaled$SOIR_desire <- rowMeans(data_scaled[,c(18:20)])
data_scaled$SOIR_full <- rowMeans(data_scaled[,c(12:20)])
```

Notes SDI: SDI equations below (some need 1 subtracted)

Items 10-12 = solitary desire, (Item10) + Item11-1 + (Item12-1) as solitary_SDI,

Items 1-8 = dyadic desire Item1 + Item2 + (Item3-1) + (Item4-1) + (Item5-1) + (Item6-1) + (Item7-1) + (Item8-1) as dyadic_SDI,

All items summed = (again those with 9-point scales -1, which are Items 3-9, 11-14) as total_SDI

```
data_scaled$SDI_solitary <- data_scaled$SDI10 + (data_scaled$SDI11-1) + (data_scaled$SDI12 -1)

data_scaled$SDI_dyadic <- data_scaled$SDI1 + data_scaled$SDI2 + (data_scaled$SDI3 -1) + (data_scaled$SDI4 -1) + (data_scaled$SDI5 -1) + (data_scaled$SDI6 -1) + (data_scaled$SDI7 -1) + (data_scaled$SDI8 -1)

data_scaled$SDI_full <- data_scaled$SDI1 + data_scaled$SDI2 + (data_scaled$SDI3 -1) + (data_scaled$SDI4 -1) + (data_scaled$SDI5 -1) + (data_scaled$SDI6 -1) + (data_scaled$SDI7 -1) + (data_scaled$SDI8 -1) + (data_scaled$SDI9 -1) + data_scaled$SDI10 + (data_scaled$SDI11 -1) + (data_scaled$SDI12 -1) + (data_scaled$SDI13 -1) + (data_scaled$SDI14 -1)


###descriptives
psych::describe(data_scaled)
```

```
##                     vars   n   mean    sd median trimmed   mad   min    max
## hm_id*                 1 283  34.59 19.53  35.00   34.63 25.20  1.00  68.00
## relationship*          2 283   1.49  0.50   1.00    1.48  0.00  1.00   2.00
## age                    3 283  22.11  3.15  21.50   21.69  2.82 18.20  31.50
## date*                  4 283  20.33 10.71  21.00   20.50 13.34  1.00  39.00
## sex*                   5 283   1.00  0.00   1.00    1.00  0.00  1.00   1.00
## ethnicity*             6 283   4.81  0.72   5.00    5.00  0.00  1.00   5.00
## sexpref*               7 283   3.88  0.52   4.00    4.00  0.00  1.00   4.00
## session                8 283   2.95  1.45   3.00    2.93  1.48  1.00   6.00
## testosterone           9 279 178.26 41.97 169.66  175.41 37.97 60.70 314.95
## cortisol              10 282   0.19  0.11   0.16    0.18  0.07  0.04   0.62
## labnotes*             11 283   1.00  0.06   1.00    1.00  0.00  1.00   2.00
## behavior1             12 257   2.27  0.91   2.00    2.21  1.48  1.00   4.00
## behavior2             13 262   2.09  0.95   2.00    1.99  1.48  1.00   5.00
## behavior3             14 262   2.24  1.15   2.00    2.11  1.48  1.00   5.00
## attitude1             15 274   3.72  1.30   4.00    3.90  1.48  1.00   5.00
## attitude2             16 273   3.18  1.33   3.00    3.23  1.48  1.00   5.00
## attitude3             17 270   3.63  1.29   4.00    3.79  1.48  1.00   5.00
## desire1               18 269   3.53  1.08   4.00    3.54  1.48  1.00   5.00
## desire2               19 270   3.05  1.01   3.00    3.06  1.48  1.00   5.00
## desire3               20 270   3.16  1.18   3.00    3.14  1.48  1.00   5.00
## trait_sexdrive        21 275   4.72  1.38   5.00    4.80  1.48  1.00   7.00
## state_sexdrive        22 274   4.29  1.57   4.00    4.35  1.48  1.00   7.00
## SDI1                  23 270   4.00  1.79   4.00    4.13  1.48  0.00   7.00
## SDI2                  24 269   4.17  1.74   4.00    4.25  1.48  0.00   7.00
## SDI3                  25 274   6.36  1.80   7.00    6.49  1.48  1.00   9.00
## SDI4                  26 275   5.37  1.80   6.00    5.44  1.48  1.00   9.00
## SDI5                  27 275   5.75  1.83   6.00    5.89  1.48  1.00   9.00
## SDI6                  28 274   6.11  1.84   6.00    6.23  1.48  1.00   9.00
## SDI7                  29 274   6.64  1.64   7.00    6.79  1.48  1.00   9.00
## SDI8                  30 274   6.09  2.10   7.00    6.24  1.48  1.00   9.00
## SDI9                  31 272   5.39  1.80   5.00    5.50  1.48  1.00   9.00
## SDI10                 32 268   3.51  1.73   4.00    3.62  1.48  0.00   7.00
## SDI11                 33 271   4.82  1.93   5.00    4.81  1.48  1.00   9.00
## SDI12                 34 271   4.31  2.27   4.00    4.27  2.97  1.00   9.00
## SDI13                 35 272   4.58  1.84   5.00    4.56  1.48  1.00   9.00
## SDI14                 36 273   3.70  1.58   4.00    3.70  1.48  1.00   8.00
## testosterone.s        37 279   0.00  0.12   0.00    0.00  0.11 -0.31   0.38
## cortisol.s            38 282   0.00  0.16  -0.02   -0.01  0.11 -0.39   0.67
## avg_testosterone      39 283 179.11 36.28 169.30  176.69 37.23 94.81 265.10
## avg_cortisol          40 283   0.19  0.08   0.18    0.19  0.06  0.07   0.41
## avg_testosterone.s    41 283   0.00  0.20  -0.06   -0.01  0.21 -0.47   0.48
## avg_cortisol.s        42 283   0.00  0.15  -0.01   -0.01  0.12 -0.25   0.44
## SOIR_behavior         43 257   2.17  0.81   2.00    2.12  0.99  1.00   4.67
## SOIR_attitude         44 270   3.52  1.15   3.67    3.63  0.99  1.00   5.00
## SOIR_desire           45 269   3.25  0.95   3.33    3.25  0.99  1.33   5.00
## SOIR_full             46 255   2.98  0.75   3.11    3.02  0.66  1.11   4.33
## SDI_solitary          47 268  10.63  5.11  11.00   10.70  5.93  0.00  23.00
## SDI_dyadic            48 267  38.55 10.04  40.00   39.33  8.90  1.00  59.00
## SDI_full              49 264  60.11 16.33  60.00   60.41 14.83 11.00  96.00
##                      range  skew kurtosis   se
## hm_id*               67.00 -0.02    -1.24 1.16
```

```
## relationship*         1.00  0.05    -2.00 0.03
## age                  13.30  1.11     0.80 0.19
## date*                38.00 -0.15    -1.16 0.64
## sex*                  0.00   NaN      NaN 0.00
## ethnicity*            4.00 -4.01    15.72 0.04
## sexpref*              3.00 -4.63    20.92 0.03
## session               5.00  0.09    -1.29 0.09
## testosterone        254.25  0.54     0.15 2.51
## cortisol              0.58  1.52     2.48 0.01
## labnotes*             1.00 16.64   276.02 0.00
## behavior1             3.00  0.28    -0.72 0.06
## behavior2             4.00  0.99     1.14 0.06
## behavior3             4.00  0.62    -0.46 0.07
## attitude1             4.00 -0.85    -0.31 0.08
## attitude2             4.00 -0.23    -1.12 0.08
## attitude3             4.00 -0.91    -0.23 0.08
## desire1               4.00 -0.23    -1.20 0.07
## desire2               4.00 -0.06    -1.01 0.06
## desire3               4.00 -0.01    -1.19 0.07
## trait_sexdrive        6.00 -0.49     0.03 0.08
## state_sexdrive        6.00 -0.27    -0.63 0.09
## SDI1                  7.00 -0.62    -0.12 0.11
## SDI2                  7.00 -0.37    -0.74 0.11
## SDI3                  8.00 -0.62    -0.08 0.11
## SDI4                  8.00 -0.41    -0.78 0.11
## SDI5                  8.00 -0.60    -0.44 0.11
## SDI6                  8.00 -0.56    -0.28 0.11
## SDI7                  8.00 -0.88     0.92 0.10
## SDI8                  8.00 -0.61    -0.38 0.13
## SDI9                  8.00 -0.44    -0.10 0.11
## SDI10                 7.00 -0.53    -0.27 0.11
## SDI11                 8.00  0.05    -0.61 0.12
## SDI12                 8.00  0.14    -1.13 0.14
## SDI13                 8.00  0.12    -0.29 0.11
## SDI14                 7.00  0.11    -0.77 0.10
## testosterone.s        0.69  0.29     0.19 0.01
## cortisol.s            1.06  1.18     2.82 0.01
## avg_testosterone    170.30  0.52    -0.01 2.16
## avg_cortisol          0.34  0.79     0.21 0.00
## avg_testosterone.s    0.95  0.52    -0.01 0.01
## avg_cortisol.s        0.68  0.79     0.21 0.01
## SOIR_behavior         3.67  0.44    -0.38 0.05
## SOIR_attitude         4.00 -0.69    -0.28 0.07
## SOIR_desire           3.67 -0.17    -1.09 0.06
## SOIR_full             3.22 -0.53    -0.60 0.05
## SDI_solitary         23.00 -0.10    -0.71 0.31
## SDI_dyadic           58.00 -0.93     1.29 0.61
## SDI_full             85.00 -0.27     0.24 1.01
```

Exclude non-straight men (n=4) to see if that changes any results

```
# set values > 3SD from the mean to NA
data_scaled2 <- data_scaled

data_scaled2<-data_scaled2[(data_scaled2$sexpref=="women"),]
```

ANALYSES

Results for LMEM Analysis for SDI

```
##full scale sdi
SDI_full_model <- lmer(SDI_full ~ 1 + testosterone.s * cortisol.s +
                        avg_testosterone.s * avg_cortisol.s +
                        (testosterone.s * cortisol.s || hm_id),
                     data = data_scaled, REML = FALSE)
```

```
## boundary (singular) fit: see ?isSingular
```

```
summary.SDI_full_model <- summary(SDI_full_model)

summary.SDI_full_model$coefficients %>%
  as.data.frame() %>%
  rownames_to_column(var = "Effect") %>%
  mutate_if(is.numeric, round, 3) %>%
  rename(p = `Pr(>|t|)`)
```

```
##                               Effect Estimate Std. Error     df t value
## 1                        (Intercept)   61.916      2.166 58.796  28.591
## 2                     testosterone.s    1.285      2.787 30.489   0.461
## 3                         cortisol.s   -2.068      2.236 11.103  -0.925
## 4                 avg_testosterone.s    2.089     11.812 59.179   0.177
## 5                     avg_cortisol.s  -17.122     14.742 60.012  -1.161
## 6          testosterone.s:cortisol.s   27.039     17.940 59.409   1.507
## 7 avg_testosterone.s:avg_cortisol.s  -74.805     52.565 58.647  -1.423
##       p
## 1 0.000
## 2 0.648
## 3 0.375
## 4 0.860
## 5 0.250
## 6 0.137
## 7 0.160
```

```r
#sdi solitary
SDI_solitary_model <- lmer(SDI_solitary ~ 1 + testosterone.s * cortisol.s +
                        avg_testosterone.s * avg_cortisol.s +
                        (testosterone.s * cortisol.s || hm_id),
                     data = data_scaled, REML = FALSE)
```

```
## boundary (singular) fit: see ?isSingular
```

```r
summary.SDI_solitary_model <- summary(SDI_solitary_model)

summary.SDI_solitary_model$coefficients %>%
  as.data.frame() %>%
  rownames_to_column(var = "Effect") %>%
  mutate_if(is.numeric, round, 3) %>%
  rename(p = `Pr(>|t|)`)
```

```
##                              Effect Estimate Std. Error      df t value
## 1                       (Intercept)   11.324      0.651  60.158  17.398
## 2                    testosterone.s   -1.647      0.928 202.934  -1.776
## 3                        cortisol.s    0.409      0.772 204.013   0.530
## 4                avg_testosterone.s    3.689      3.550  60.532   1.039
## 5                    avg_cortisol.s   -4.056      4.441  61.884  -0.913
## 6         testosterone.s:cortisol.s    2.245      6.267 207.805   0.358
## 7 avg_testosterone.s:avg_cortisol.s  -28.445     15.801  60.045  -1.800
##       p
## 1 0.000
## 2 0.077
## 3 0.597
## 4 0.303
## 5 0.365
## 6 0.720
## 7 0.077
```

```r
#sdi dyadic
SDI_dyadic_model <- lmer(SDI_dyadic ~ 1 + testosterone.s * cortisol.s +
                        avg_testosterone.s * avg_cortisol.s +
                        (testosterone.s * cortisol.s || hm_id),
                     data = data_scaled, REML = FALSE)
```

```
## boundary (singular) fit: see ?isSingular
```

```
summary.SDI_dyadic_model <- summary(SDI_dyadic_model)

summary.SDI_dyadic_model$coefficients %>%
  as.data.frame() %>%
  rownames_to_column(var = "Effect") %>%
  mutate_if(is.numeric, round, 3) %>%
  rename(p = `Pr(>|t|)`)
```

```
##                              Effect Estimate Std. Error      df t value
## 1                       (Intercept)   39.280      1.322  58.594  29.710
## 2                    testosterone.s    3.961      2.351  30.147   1.685
## 3                        cortisol.s   -1.182      1.673 190.276  -0.706
## 4                avg_testosterone.s   -0.754      7.269  59.111  -0.104
## 5                    avg_cortisol.s  -10.168      9.083  60.291  -1.119
## 6         testosterone.s:cortisol.s   22.269     13.633 201.732   1.633
## 7 avg_testosterone.s:avg_cortisol.s  -38.649     32.345  58.347  -1.195
##       p
## 1 0.000
## 2 0.102
## 3 0.481
## 4 0.918
## 5 0.267
## 6 0.104
## 7 0.237
```

Results for LMEM Analysis for SOI_R

```
#SOIR full scale
SOIR_full_model <- lmer(SOIR_full ~ 1 + testosterone.s * cortisol.s +
                        avg_testosterone.s * avg_cortisol.s +
                        (testosterone.s * cortisol.s || hm_id),
                     data = data_scaled, REML = FALSE)
```

```
## Warning in checkConv(attr(opt, "derivs"), opt$par, ctrl =
## control$checkConv, : unable to evaluate scaled gradient
```

```
## Warning in checkConv(attr(opt, "derivs"), opt$par, ctrl =
## control$checkConv, : Model failed to converge: degenerate Hessian with 1
## negative eigenvalues
```

```
## Warning: Model failed to converge with 1 negative eigenvalue: -3.2e-02
```

```
summary.SOIR_full_model <- summary(SOIR_full_model)

summary.SOIR_full_model$coefficients %>%
  as.data.frame() %>%
  rownames_to_column(var = "Effect") %>%
  mutate_if(is.numeric, round, 3) %>%
  rename(p = `Pr(>|t|)`)
```

```
##                              Effect Estimate Std. Error      df t value
## 1                       (Intercept)    3.108      0.096  57.056  32.461
## 2                    testosterone.s   -0.050      0.131  37.534  -0.384
## 3                        cortisol.s   -0.126      0.119  28.839  -1.061
## 4                avg_testosterone.s    0.912      0.509  57.403   1.791
## 5                    avg_cortisol.s   -0.201      0.641  58.431  -0.314
## 6         testosterone.s:cortisol.s    0.473      0.874 154.809   0.542
## 7 avg_testosterone.s:avg_cortisol.s   -6.174      2.269  56.976  -2.721
##       p
## 1 0.000
## 2 0.703
## 3 0.297
## 4 0.079
## 5 0.755
## 6 0.589
## 7 0.009
```

```
#SOIR behavior
SOIR_behavior_model <- lmer(SOIR_behavior ~ 1 + testosterone.s * cortisol.s +
                        avg_testosterone.s * avg_cortisol.s +
                        (testosterone.s * cortisol.s || hm_id),
                     data = data_scaled, REML = FALSE)
```

```
## boundary (singular) fit: see ?isSingular
```

```
summary.SOIR_behavior_model <- summary(SOIR_behavior_model)

summary.SOIR_behavior_model$coefficients %>%
  as.data.frame() %>%
  rownames_to_column(var = "Effect") %>%
  mutate_if(is.numeric, round, 3) %>%
  rename(p = `Pr(>|t|)`)
```

```
##                                Effect Estimate Std. Error      df t value
## 1                        (Intercept)    2.208      0.114  57.101  19.422
## 2                     testosterone.s   -0.139      0.139 192.790  -1.000
## 3                         cortisol.s   -0.043      0.116 144.071  -0.366
## 4                 avg_testosterone.s    0.756      0.610  57.327   1.239
## 5                     avg_cortisol.s    0.291      0.767  58.387   0.380
## 6          testosterone.s:cortisol.s    1.703      0.999  14.449   1.706
## 7 avg_testosterone.s:avg_cortisol.s   -0.324      2.717  56.984  -0.119
##       p
## 1 0.000
## 2 0.318
## 3 0.715
## 4 0.220
## 5 0.705
## 6 0.109
## 7 0.906
```

```
#SOIR attitude
SOIR_attitude_model <- lmer(SOIR_attitude ~ 1 + testosterone.s * cortisol.s +
                        avg_testosterone.s * avg_cortisol.s +
                        (testosterone.s * cortisol.s || hm_id),
                     data = data_scaled, REML = FALSE)
```

```
## Warning in checkConv(attr(opt, "derivs"), opt$par, ctrl =
## control$checkConv, : unable to evaluate scaled gradient
```

```
## Warning in checkConv(attr(opt, "derivs"), opt$par, ctrl =
## control$checkConv, : Model failed to converge: degenerate Hessian with 1
## negative eigenvalues
```

```
## Warning: Model failed to converge with 1 negative eigenvalue: -2.0e-03
```

```
summary.SOIR_attitude_model <- summary(SOIR_attitude_model)

summary.SOIR_attitude_model$coefficients %>%
  as.data.frame() %>%
  rownames_to_column(var = "Effect") %>%
  mutate_if(is.numeric, round, 3) %>%
  rename(p = `Pr(>|t|)`)
```

```
##                                Effect Estimate Std. Error      df t value
## 1                        (Intercept)    3.761      0.136  60.653  27.697
## 2                     testosterone.s    0.116      0.256  36.505   0.452
## 3                         cortisol.s    0.094      0.215  23.516   0.437
## 4                 avg_testosterone.s    1.236      0.740  61.020   1.671
## 5                     avg_cortisol.s   -0.293      0.927  62.556  -0.317
## 6          testosterone.s:cortisol.s   -1.504      1.537 148.323  -0.979
## 7 avg_testosterone.s:avg_cortisol.s  -11.834      3.296  60.509  -3.590
##       p
## 1 0.000
## 2 0.654
## 3 0.666
## 4 0.100
## 5 0.753
## 6 0.329
## 7 0.001
```

```
#SOIR desire
SOIR_desire_model <- lmer(SOIR_desire ~ 1 + testosterone.s * cortisol.s +
                        avg_testosterone.s * avg_cortisol.s +
                        (testosterone.s * cortisol.s || hm_id),
                     data = data_scaled, REML = FALSE)
```

```
## Warning in checkConv(attr(opt, "derivs"), opt$par, ctrl =
## control$checkConv, : unable to evaluate scaled gradient
```

```
## Warning in checkConv(attr(opt, "derivs"), opt$par, ctrl =
## control$checkConv, : Model failed to converge: degenerate Hessian with 1
## negative eigenvalues
```

```
## Warning: Model failed to converge with 1 negative eigenvalue: -3.8e-02
```

```
summary.SOIR_desire_model <- summary(SOIR_desire_model)

summary.SOIR_desire_model$coefficients %>%
  as.data.frame() %>%
  rownames_to_column(var = "Effect") %>%
  mutate_if(is.numeric, round, 3) %>%
  rename(p = `Pr(>|t|)`)
```

```
##                               Effect Estimate Std. Error      df t value
## 1                        (Intercept)    3.374      0.116  60.286  29.174
## 2                     testosterone.s   -0.003      0.207 200.316  -0.013
## 3                         cortisol.s   -0.473      0.237  44.869  -1.995
## 4                 avg_testosterone.s    0.892      0.631  60.940   1.414
## 5                     avg_cortisol.s   -0.716      0.792  62.679  -0.904
## 6          testosterone.s:cortisol.s    1.425      1.527 200.029   0.933
## 7 avg_testosterone.s:avg_cortisol.s   -6.425      2.806  60.044  -2.290
##       p
## 1 0.000
## 2 0.989
## 3 0.052
## 4 0.163
## 5 0.369
## 6 0.352
## 7 0.026
```

ANALYSES PART 2

Do all analyses again for straight people only

Results for LMEM Analysis for SDI

```
##full scale sdi
SDI_full_model2 <- lmer(SDI_full ~ 1 + testosterone.s * cortisol.s +
                        avg_testosterone.s * avg_cortisol.s +
                        (testosterone.s * cortisol.s || hm_id),
                     data = data_scaled2, REML = FALSE)

summary.SDI_full_model2 <- summary(SDI_full_model2)

summary.SDI_full_model2$coefficients %>%
  as.data.frame() %>%
  rownames_to_column(var = "Effect") %>%
  mutate_if(is.numeric, round, 3) %>%
  rename(p = `Pr(>|t|)`)
```

```
##                              Effect Estimate Std. Error     df t value
## 1                       (Intercept)   62.776      2.124 55.925  29.563
## 2                    testosterone.s    1.678      2.876 29.906   0.584
## 3                        cortisol.s   -2.449      2.291  9.979  -1.069
## 4                avg_testosterone.s    0.263     11.297 56.460   0.023
## 5                    avg_cortisol.s  -14.751     14.430 57.077  -1.022
## 6         testosterone.s:cortisol.s   25.871     18.636 58.717   1.388
## 7 avg_testosterone.s:avg_cortisol.s  -84.068     50.115 55.945  -1.677
##       p
## 1 0.000
## 2 0.564
## 3 0.310
## 4 0.982
## 5 0.311
## 6 0.170
## 7 0.099
```

```
#sdi solitary
SDI_solitary_model2 <- lmer(SDI_solitary ~ 1 + testosterone.s * cortisol.s +
                        avg_testosterone.s * avg_cortisol.s +
                        (testosterone.s * cortisol.s || hm_id),
                     data = data_scaled2, REML = FALSE)
```

```
## boundary (singular) fit: see ?isSingular
```

```
summary.SDI_solitary_model2 <- summary(SDI_solitary_model2)

summary.SDI_solitary_model2$coefficients %>%
  as.data.frame() %>%
  rownames_to_column(var = "Effect") %>%
  mutate_if(is.numeric, round, 3) %>%
  rename(p = `Pr(>|t|)`)
```

```
##                              Effect Estimate Std. Error      df t value
## 1                       (Intercept)   11.335      0.684  56.175  16.566
## 2                    testosterone.s   -1.418      0.939 194.816  -1.510
## 3                        cortisol.s    0.583      0.792 195.899   0.736
## 4                avg_testosterone.s    3.293      3.640  56.697   0.905
## 5                    avg_cortisol.s   -3.408      4.656  57.629  -0.732
## 6         testosterone.s:cortisol.s    1.221      6.507 199.586   0.188
## 7 avg_testosterone.s:avg_cortisol.s  -29.426     16.155  56.297  -1.821
##       p
## 1 0.000
## 2 0.133
## 3 0.462
## 4 0.369
## 5 0.467
## 6 0.851
## 7 0.074
```

```
#sdi dyadic
SDI_dyadic_model2 <- lmer(SDI_dyadic ~ 1 + testosterone.s * cortisol.s +
                        avg_testosterone.s * avg_cortisol.s +
                        (testosterone.s * cortisol.s || hm_id),
                     data = data_scaled2, REML = FALSE)
```

```
## boundary (singular) fit: see ?isSingular
```

```
summary.SDI_dyadic_model2 <- summary(SDI_dyadic_model2)

summary.SDI_dyadic_model2$coefficients %>%
  as.data.frame() %>%
  rownames_to_column(var = "Effect") %>%
  mutate_if(is.numeric, round, 3) %>%
  rename(p = `Pr(>|t|)`)
```

```
##                              Effect Estimate Std. Error      df t value
## 1                       (Intercept)   39.907      1.266  56.804  31.512
## 2                    testosterone.s    4.006      2.365  28.512   1.694
## 3                        cortisol.s   -1.747      1.721 188.755  -1.015
## 4                avg_testosterone.s   -2.159      6.799  57.568  -0.318
## 5                    avg_cortisol.s   -8.194      8.692  58.477  -0.943
## 6         testosterone.s:cortisol.s   23.292     14.175 198.260   1.643
## 7 avg_testosterone.s:avg_cortisol.s  -45.025     30.154  56.784  -1.493
##       p
## 1 0.000
## 2 0.101
## 3 0.311
## 4 0.752
## 5 0.350
## 6 0.102
## 7 0.141
```

Results for LMEM Analysis for SOI_R

```
#SOIR full scale
SOIR_full_model2 <- lmer(SOIR_full ~ 1 + testosterone.s * cortisol.s +
                        avg_testosterone.s * avg_cortisol.s +
                        (testosterone.s * cortisol.s || hm_id),
                     data = data_scaled2, REML = FALSE)
```

```
## Warning in checkConv(attr(opt, "derivs"), opt$par, ctrl =
## control$checkConv, : Model failed to converge with max|grad| = 0.00252753
## (tol = 0.002, component 1)
```

```
summary.SOIR_full_model2 <- summary(SOIR_full_model2)

summary.SOIR_full_model2$coefficients %>%
  as.data.frame() %>%
  rownames_to_column(var = "Effect") %>%
  mutate_if(is.numeric, round, 3) %>%
  rename(p = `Pr(>|t|)`)
```

```
##                               Effect Estimate Std. Error     df t value
## 1                        (Intercept)    3.099      0.098 54.948  31.487
## 2                     testosterone.s   -0.061      0.132 36.574  -0.464
## 3                         cortisol.s   -0.132      0.120 27.724  -1.104
## 4                 avg_testosterone.s    0.917      0.520 55.401   1.765
## 5                     avg_cortisol.s   -0.148      0.664 56.120  -0.222
## 6          testosterone.s:cortisol.s    0.674      0.945 20.104   0.714
## 7 avg_testosterone.s:avg_cortisol.s   -6.195      2.304 54.983  -2.688
##       p
## 1 0.000
## 2 0.646
## 3 0.279
## 4 0.083
## 5 0.825
## 6 0.484
## 7 0.009
```

```
#SOIR behavior
SOIR_behavior_model2 <- lmer(SOIR_behavior ~ 1 + testosterone.s * cortisol.s +
                        avg_testosterone.s * avg_cortisol.s +
                        (testosterone.s * cortisol.s || hm_id),
                     data = data_scaled2, REML = FALSE)
```

```
## boundary (singular) fit: see ?isSingular
```

```
summary.SOIR_behavior_model2 <- summary(SOIR_behavior_model2)

summary.SOIR_behavior_model2$coefficients %>%
  as.data.frame() %>%
  rownames_to_column(var = "Effect") %>%
  mutate_if(is.numeric, round, 3) %>%
  rename(p = `Pr(>|t|)`)
```

```
##                               Effect Estimate Std. Error      df t value
## 1                        (Intercept)    2.195      0.112  53.964  19.545
## 2                     testosterone.s   -0.143      0.141 188.610  -1.017
## 3                         cortisol.s   -0.041      0.118 141.171  -0.351
## 4                 avg_testosterone.s    0.767      0.593  54.406   1.294
## 5                     avg_cortisol.s    0.553      0.758  55.248   0.729
## 6          testosterone.s:cortisol.s    1.683      1.004  14.226   1.677
## 7 avg_testosterone.s:avg_cortisol.s   -0.778      2.630  54.067  -0.296
##       p
## 1 0.000
## 2 0.310
## 3 0.726
## 4 0.201
## 5 0.469
## 6 0.115
## 7 0.768
```

```
#SOIR attitude
SOIR_attitude_model2 <- lmer(SOIR_attitude ~ 1 + testosterone.s * cortisol.s +
                        avg_testosterone.s * avg_cortisol.s +
                        (testosterone.s * cortisol.s || hm_id),
                     data = data_scaled2, REML = FALSE)
```

```
## Warning in checkConv(attr(opt, "derivs"), opt$par, ctrl =
## control$checkConv, : Model failed to converge with max|grad| = 0.0174095
## (tol = 0.002, component 1)
```

```
summary.SOIR_attitude_model2 <- summary(SOIR_attitude_model2)

summary.SOIR_attitude_model2$coefficients %>%
  as.data.frame() %>%
  rownames_to_column(var = "Effect") %>%
  mutate_if(is.numeric, round, 3) %>%
  rename(p = `Pr(>|t|)`)
```

```
##                              Effect Estimate Std. Error     df t value
## 1                       (Intercept)    3.738      0.141 57.762  26.516
## 2                    testosterone.s    0.095      0.265 35.385   0.358
## 3                        cortisol.s    0.104      0.227 24.313   0.457
## 4                avg_testosterone.s    1.197      0.756 58.146   1.584
## 5                    avg_cortisol.s   -0.135      0.967 59.302  -0.140
## 6         testosterone.s:cortisol.s   -1.577      1.668 16.481  -0.946
## 7 avg_testosterone.s:avg_cortisol.s  -11.895      3.356 57.697  -3.544
##       p
## 1 0.000
## 2 0.722
## 3 0.652
## 4 0.119
## 5 0.889
## 6 0.358
## 7 0.001
```

```
#SOIR desire
SOIR_desire_model2 <- lmer(SOIR_desire ~ 1 + testosterone.s * cortisol.s +
                        avg_testosterone.s * avg_cortisol.s +
                        (testosterone.s * cortisol.s || hm_id),
                     data = data_scaled2, REML = FALSE)
```

```
## boundary (singular) fit: see ?isSingular
```

```
summary.SOIR_desire_model2 <- summary(SOIR_desire_model2)

summary.SOIR_desire_model2$coefficients %>%
  as.data.frame() %>%
  rownames_to_column(var = "Effect") %>%
  mutate_if(is.numeric, round, 3) %>%
  rename(p = `Pr(>|t|)`)
```

```
##                                Effect Estimate Std. Error      df t value
## 1                         (Intercept)    3.391      0.118  57.218  28.817
## 2                      testosterone.s   -0.075      0.215 194.366  -0.351
## 3                          cortisol.s   -0.505      0.248  45.424  -2.040
## 4                  avg_testosterone.s    0.889      0.632  58.024   1.407
## 5                      avg_cortisol.s   -1.010      0.809  59.220  -1.248
## 6           testosterone.s:cortisol.s    2.085      1.838  18.539   1.135
## 7 avg_testosterone.s:avg_cortisol.s   -6.100      2.800  57.097  -2.178
##       p
## 1 0.000
## 2 0.726
## 3 0.047
## 4 0.165
## 5 0.217
## 6 0.271
## 7 0.034
```

Additional analyses as requested in the review process

```
#Cronbachs alpha for the scales

#first SOI-R
data_soibehavior <- data[,c(12:14)]
data_soiattitude <- data[,c(15:17)]
data_soidesire <- data[,c(18:20)]
data_soifull <- data[,c(12:20)]

psych::alpha(data_soidesire)
```

```
##
## Reliability analysis
## Call: psych::alpha(x = data_soidesire)
##
##   raw_alpha std.alpha G6(smc) average_r S/N   ase mean   sd median_r
##       0.84      0.84    0.78      0.64 5.3 0.016  3.2 0.95     0.61
##
##  lower alpha upper     95% confidence boundaries
## 0.81 0.84 0.87
##
##  Reliability if an item is dropped:
##         raw_alpha std.alpha G6(smc) average_r S/N alpha se var.r med.r
## desire1      0.75      0.75    0.61      0.61 3.1    0.029    NA  0.61
## desire2      0.82      0.82    0.70      0.70 4.7    0.021    NA  0.70
## desire3      0.75      0.75    0.60      0.60 3.1    0.029    NA  0.60
##
##  Item statistics
##           n raw.r std.r r.cor r.drop mean  sd
## desire1 269  0.88  0.88  0.80   0.73  3.5 1.1
## desire2 270  0.83  0.85  0.71   0.66  3.1 1.0
## desire3 270  0.89  0.88  0.80   0.73  3.2 1.2
##
## Non missing response frequency for each item
##            1    2    3    4    5 miss
## desire1 0.00 0.25 0.16 0.38 0.20 0.05
## desire2 0.04 0.31 0.24 0.35 0.05 0.05
## desire3 0.06 0.33 0.16 0.32 0.14 0.05
```

```
psych::alpha(data_soiattitude)
```

```
##
## Reliability analysis
## Call: psych::alpha(x = data_soiattitude)
##
##   raw_alpha std.alpha G6(smc) average_r S/N   ase mean  sd median_r
##       0.85      0.85    0.79      0.65 5.5 0.016  3.5 1.1     0.64
##
##  lower alpha upper     95% confidence boundaries
## 0.82 0.85 0.88
##
##  Reliability if an item is dropped:
##           raw_alpha std.alpha G6(smc) average_r S/N alpha se var.r med.r
## attitude1      0.78      0.78    0.64      0.64 3.5    0.026    NA  0.64
## attitude2      0.76      0.76    0.61      0.61 3.2    0.028    NA  0.61
## attitude3      0.82      0.82    0.69      0.69 4.4    0.022    NA  0.69
##
##  Item statistics
##             n raw.r std.r r.cor r.drop mean  sd
## attitude1 274  0.88  0.88  0.79   0.72  3.7 1.3
## attitude2 273  0.89  0.89  0.81   0.74  3.2 1.3
## attitude3 270  0.85  0.86  0.74   0.68  3.6 1.3
##
## Non missing response frequency for each item
##              1    2    3    4    5 miss
## attitude1 0.12 0.04 0.20 0.28 0.35 0.03
## attitude2 0.15 0.17 0.22 0.27 0.19 0.04
## attitude3 0.13 0.05 0.14 0.41 0.27 0.05
```

```
psych::alpha(data_soibehavior)
```

```
##
## Reliability analysis
## Call: psych::alpha(x = data_soibehavior)
##
##   raw_alpha std.alpha G6(smc) average_r S/N   ase mean   sd median_r
##       0.77      0.77    0.71      0.53 3.4 0.023  2.2 0.86     0.51
##
##  lower alpha upper     95% confidence boundaries
## 0.73 0.77 0.82
##
##  Reliability if an item is dropped:
##           raw_alpha std.alpha G6(smc) average_r S/N alpha se var.r med.r
## behavior1      0.78      0.79    0.65      0.65 3.7    0.026    NA  0.65
## behavior2      0.66      0.67    0.51      0.51 2.0    0.039    NA  0.51
## behavior3      0.61      0.61    0.44      0.44 1.5    0.047    NA  0.44
##
##  Item statistics
##             n raw.r std.r r.cor r.drop mean   sd
## behavior1 257  0.78  0.78  0.58   0.52  2.3 0.91
## behavior2 262  0.85  0.84  0.73   0.64  2.1 0.95
## behavior3 262  0.88  0.87  0.78   0.68  2.2 1.15
##
## Non missing response frequency for each item
##              1    2    3    4    5 miss
## behavior1 0.21 0.42 0.27 0.11 0.00 0.09
## behavior2 0.28 0.45 0.20 0.03 0.03 0.07
## behavior3 0.34 0.27 0.26 0.09 0.05 0.07
```

```
psych::alpha(data_soifull)
```

```
##
## Reliability analysis
## Call: psych::alpha(x = data_soifull)
##
##   raw_alpha std.alpha G6(smc) average_r S/N   ase mean   sd median_r
##       0.83      0.83    0.87      0.35 4.9 0.015    3 0.76      0.3
##
##  lower alpha upper     95% confidence boundaries
## 0.8 0.83 0.86
##
##  Reliability if an item is dropped:
##           raw_alpha std.alpha G6(smc) average_r S/N alpha se var.r med.r
## behavior1      0.83      0.83    0.87      0.38 4.9    0.015 0.029  0.33
## behavior2      0.82      0.81    0.85      0.35 4.3    0.016 0.034  0.28
## behavior3      0.82      0.82    0.85      0.36 4.4    0.016 0.033  0.31
## attitude1      0.81      0.81    0.85      0.35 4.3    0.017 0.030  0.31
## attitude2      0.80      0.81    0.85      0.34 4.1    0.018 0.030  0.29
## attitude3      0.80      0.80    0.85      0.33 4.0    0.018 0.033  0.27
## desire1        0.80      0.80    0.84      0.34 4.1    0.017 0.029  0.28
## desire2        0.82      0.82    0.86      0.36 4.5    0.016 0.031  0.33
## desire3        0.82      0.82    0.85      0.36 4.5    0.016 0.026  0.31
##
##  Item statistics
##             n raw.r std.r r.cor r.drop mean   sd
## behavior1 257  0.49  0.52  0.43   0.38  2.3 0.91
## behavior2 262  0.63  0.66  0.61   0.53  2.1 0.95
## behavior3 262  0.61  0.63  0.58   0.49  2.2 1.15
## attitude1 274  0.69  0.67  0.64   0.58  3.7 1.30
## attitude2 273  0.72  0.70  0.67   0.62  3.2 1.33
## attitude3 270  0.76  0.74  0.70   0.66  3.6 1.29
## desire1   269  0.73  0.73  0.71   0.63  3.5 1.08
## desire2   270  0.60  0.61  0.55   0.48  3.1 1.01
## desire3   270  0.60  0.61  0.57   0.48  3.2 1.18
##
## Non missing response frequency for each item
##              1    2    3    4    5 miss
## behavior1 0.21 0.42 0.27 0.11 0.00 0.09
## behavior2 0.28 0.45 0.20 0.03 0.03 0.07
## behavior3 0.34 0.27 0.26 0.09 0.05 0.07
## attitude1 0.12 0.04 0.20 0.28 0.35 0.03
## attitude2 0.15 0.17 0.22 0.27 0.19 0.04
## attitude3 0.13 0.05 0.14 0.41 0.27 0.05
## desire1   0.00 0.25 0.16 0.38 0.20 0.05
## desire2   0.04 0.31 0.24 0.35 0.05 0.05
## desire3   0.06 0.33 0.16 0.32 0.14 0.05
```

```
#then SDI-2
SDI_solitary <- data[,c(32,33,34)]
SDI_dyadic <- data[,c(23:30)]
SDI_full <- data[,c(23:36)]

psych::alpha(SDI_solitary)
```

```
##
## Reliability analysis
## Call: psych::alpha(x = SDI_solitary)
##
##   raw_alpha std.alpha G6(smc) average_r S/N   ase mean  sd median_r
##       0.82      0.82    0.76       0.6 4.6 0.018  4.2 1.7     0.57
##
##  lower alpha upper     95% confidence boundaries
## 0.78 0.82 0.85
##
##  Reliability if an item is dropped:
##       raw_alpha std.alpha G6(smc) average_r S/N alpha se var.r med.r
## SDI10      0.81      0.81    0.69      0.69 4.4    0.022    NA  0.69
## SDI11      0.71      0.73    0.57      0.57 2.6    0.033    NA  0.57
## SDI12      0.71      0.71    0.55      0.55 2.5    0.034    NA  0.55
##
##  Item statistics
##         n raw.r std.r r.cor r.drop mean  sd
## SDI10 268  0.80  0.82  0.67   0.61  3.5 1.7
## SDI11 271  0.87  0.87  0.78   0.71  4.8 1.9
## SDI12 271  0.90  0.88  0.79   0.72  4.3 2.3
```

```
psych::alpha(SDI_dyadic)
```

```
##
## Reliability analysis
## Call: psych::alpha(x = SDI_dyadic)
##
##   raw_alpha std.alpha G6(smc) average_r S/N   ase mean  sd median_r
##       0.84      0.84    0.88       0.4 5.3 0.015  5.6 1.2     0.36
##
##  lower alpha upper     95% confidence boundaries
## 0.81 0.84 0.87
##
##  Reliability if an item is dropped:
##      raw_alpha std.alpha G6(smc) average_r S/N alpha se var.r med.r
## SDI1      0.81      0.81    0.85      0.39 4.4    0.017 0.033  0.36
## SDI2      0.83      0.84    0.87      0.42 5.1    0.016 0.030  0.42
## SDI3      0.80      0.81    0.85      0.37 4.1    0.018 0.034  0.32
## SDI4      0.82      0.83    0.84      0.41 4.8    0.016 0.032  0.37
## SDI5      0.84      0.84    0.86      0.44 5.4    0.014 0.025  0.45
## SDI6      0.83      0.83    0.87      0.42 5.1    0.016 0.035  0.42
## SDI7      0.79      0.79    0.83      0.35 3.8    0.019 0.027  0.32
## SDI8      0.82      0.82    0.87      0.40 4.6    0.017 0.036  0.37
##
##  Item statistics
##        n raw.r std.r r.cor r.drop mean  sd
## SDI1 270  0.73  0.74  0.71   0.64  4.0 1.8
## SDI2 269  0.59  0.61  0.54   0.47  4.2 1.7
## SDI3 274  0.79  0.79  0.77   0.71  6.4 1.8
## SDI4 275  0.66  0.65  0.62   0.53  5.4 1.8
## SDI5 275  0.55  0.55  0.50   0.40  5.7 1.8
## SDI6 274  0.60  0.61  0.53   0.47  6.1 1.8
## SDI7 274  0.86  0.86  0.87   0.80  6.6 1.6
## SDI8 274  0.71  0.70  0.64   0.58  6.1 2.1
```

```
psych::alpha(SDI_full)
```

```
##
## Reliability analysis
## Call: psych::alpha(x = SDI_full)
##
##   raw_alpha std.alpha G6(smc) average_r S/N  ase mean  sd median_r
##       0.88      0.88    0.93      0.35 7.7 0.01  5.1 1.2     0.32
##
##  lower alpha upper     95% confidence boundaries
## 0.86 0.88 0.9
##
##  Reliability if an item is dropped:
##       raw_alpha std.alpha G6(smc) average_r S/N alpha se var.r med.r
## SDI1       0.87      0.87    0.92      0.34 6.8    0.011 0.030  0.32
## SDI2       0.88      0.88    0.92      0.36 7.3    0.011 0.031  0.33
## SDI3       0.87      0.87    0.92      0.35 6.9    0.011 0.029  0.32
## SDI4       0.88      0.88    0.91      0.36 7.3    0.011 0.031  0.34
## SDI5       0.88      0.89    0.92      0.37 7.7    0.010 0.028  0.35
## SDI6       0.88      0.88    0.93      0.37 7.6    0.010 0.029  0.35
## SDI7       0.87      0.87    0.91      0.34 6.6    0.012 0.027  0.32
## SDI8       0.88      0.88    0.92      0.35 7.1    0.011 0.031  0.33
## SDI9       0.87      0.88    0.92      0.35 7.1    0.011 0.029  0.32
## SDI10      0.88      0.89    0.93      0.38 7.8    0.010 0.025  0.35
## SDI11      0.87      0.87    0.92      0.35 7.0    0.011 0.029  0.32
## SDI12      0.88      0.88    0.92      0.35 7.1    0.011 0.030  0.32
## SDI13      0.87      0.87    0.92      0.34 6.8    0.011 0.030  0.32
## SDI14      0.88      0.88    0.92      0.35 7.1    0.011 0.032  0.32
##
##  Item statistics
##         n raw.r std.r r.cor r.drop mean  sd
## SDI1  270  0.71  0.72  0.71   0.66  4.0 1.8
## SDI2  269  0.59  0.60  0.57   0.51  4.2 1.7
## SDI3  274  0.71  0.72  0.70   0.65  6.4 1.8
## SDI4  275  0.60  0.60  0.59   0.52  5.4 1.8
## SDI5  275  0.47  0.47  0.44   0.38  5.7 1.8
## SDI6  274  0.50  0.50  0.45   0.40  6.1 1.8
## SDI7  274  0.80  0.80  0.80   0.75  6.6 1.6
## SDI8  274  0.65  0.64  0.62   0.57  6.1 2.1
## SDI9  272  0.65  0.66  0.64   0.58  5.4 1.8
## SDI10 268  0.46  0.45  0.41   0.36  3.5 1.7
## SDI11 271  0.69  0.67  0.66   0.61  4.8 1.9
## SDI12 271  0.67  0.65  0.63   0.58  4.3 2.3
## SDI13 272  0.74  0.74  0.73   0.69  4.6 1.8
## SDI14 273  0.62  0.64  0.60   0.56  3.7 1.6
```

```r
#Correlation matrix for all relevant variables

#scale all variables first to bring them to the same scale

data_scaled_cor <- data_scaled
data_scaled_cor$SOIR_behavior.s <- scale(data_scaled_cor$SOIR_behavior)
data_scaled_cor$SOIR_attitude.s <- scale(data_scaled_cor$SOIR_attitude)
data_scaled_cor$SOIR_desire.s <- scale(data_scaled_cor$SOIR_desire)
data_scaled_cor$SOIR_full.s <- scale(data_scaled_cor$SOIR_full)
data_scaled_cor$SDI_solitary.s <- scale(data_scaled_cor$SDI_solitary)
data_scaled_cor$SDI_dyadic.s <- scale(data_scaled_cor$SDI_dyadic)
data_scaled_cor$SDI_full.s <- scale(data_scaled_cor$SDI_full)

#names(data_scaled_cor)

cor_data <- data_scaled_cor[,c(37,38,41,42,50:56)]

cor(cor_data, use = "pairwise.complete.obs")
```

```
##                     testosterone.s    cortisol.s avg_testosterone.s
## testosterone.s       1.000000e+00  2.578466e-01      -6.169168e-17
## cortisol.s           2.578466e-01  1.000000e+00      -1.407057e-17
## avg_testosterone.s  -6.169168e-17 -1.407057e-17       1.000000e+00
## avg_cortisol.s      -1.450170e-16 -7.790847e-18       5.370004e-01
## SOIR_behavior.s     -2.275679e-02 -1.323380e-02       2.571439e-01
## SOIR_attitude.s      8.176030e-03  7.372913e-03       7.528622e-03
## SOIR_desire.s       -2.434361e-02 -7.457555e-02       2.419963e-02
## SOIR_full.s         -2.228729e-02 -3.191351e-02       1.129076e-01
## SDI_solitary.s      -3.998329e-02 -5.273162e-03      -1.559370e-02
## SDI_dyadic.s         4.554488e-02 -1.898856e-03      -1.681698e-01
## SDI_full.s           8.200642e-03 -1.973151e-02      -1.557184e-01
##                     avg_cortisol.s SOIR_behavior.s SOIR_attitude.s
## testosterone.s      -1.450170e-16     -0.02275679     0.008176030
## cortisol.s          -7.790847e-18     -0.01323380     0.007372913
## avg_testosterone.s   5.370004e-01      0.25714394     0.007528622
## avg_cortisol.s       1.000000e+00      0.15976094    -0.136189891
## SOIR_behavior.s      1.597609e-01      1.00000000     0.406564026
## SOIR_attitude.s     -1.361899e-01      0.40656403     1.000000000
## SOIR_desire.s       -1.146684e-01      0.25869555     0.409897761
## SOIR_full.s         -6.090962e-02      0.68098169     0.845557429
## SDI_solitary.s      -1.337217e-01      0.10585670     0.324664945
## SDI_dyadic.s        -3.049896e-01      0.38233735     0.453113328
## SDI_full.s          -2.840101e-01      0.34821981     0.479378984
##                     SOIR_desire.s SOIR_full.s SDI_solitary.s SDI_dyadic.s
## testosterone.s        -0.02434361 -0.02228729   -0.039983288  0.045544882
## cortisol.s            -0.07457555 -0.03191351   -0.005273162 -0.001898856
## avg_testosterone.s     0.02419963  0.11290763   -0.015593699 -0.168169810
## avg_cortisol.s        -0.11466842 -0.06090962   -0.133721698 -0.304989587
## SOIR_behavior.s        0.25869555  0.68098169    0.105856696  0.382337354
## SOIR_attitude.s        0.40989776  0.84555743    0.324664945  0.453113328
## SOIR_desire.s          1.00000000  0.74341877    0.257381621  0.383806796
## SOIR_full.s            0.74341877  1.00000000    0.320323589  0.537031914
## SDI_solitary.s         0.25738162  0.32032359    1.000000000  0.423063989
## SDI_dyadic.s           0.38380680  0.53703191    0.423063989  1.000000000
## SDI_full.s             0.38713756  0.53964411    0.717170502  0.915848137
##                       SDI_full.s
## testosterone.s       0.008200642
## cortisol.s          -0.019731512
## avg_testosterone.s -0.155718439
## avg_cortisol.s      -0.284010139
## SOIR_behavior.s      0.348219811
## SOIR_attitude.s      0.479378984
## SOIR_desire.s        0.387137558
## SOIR_full.s          0.539644109
## SDI_solitary.s       0.717170502
## SDI_dyadic.s         0.915848137
## SDI_full.s           1.000000000
```

```
#single correlations to get p-values and 95% CIs (results identical when computing
correlation matrix with all variables at once)
cor.test(data_scaled_cor$testosterone.s, data_scaled_cor$cortisol.s, na.rm = TRUE)
```

```
##
##  Pearson's product-moment correlation
##
## data:  data_scaled_cor$testosterone.s and data_scaled_cor$cortisol.s
## t = 4.4336, df = 276, p-value = 1.339e-05
## alternative hypothesis: true correlation is not equal to 0
## 95 percent confidence interval:
##  0.1445896 0.3644349
## sample estimates:
##       cor
## 0.2578466
```

```
cor.test(data_scaled_cor$testosterone.s, data_scaled_cor$avg_testosterone.s, na.rm
= TRUE)
```

```
##
##  Pearson's product-moment correlation
##
## data:  data_scaled_cor$testosterone.s and data_scaled_cor$avg_testosterone.s
## t = -1.0268e-15, df = 277, p-value = 1
## alternative hypothesis: true correlation is not equal to 0
## 95 percent confidence interval:
##  -0.1174317  0.1174317
## sample estimates:
##           cor
## -6.169544e-17
```

```
cor.test(data_scaled_cor$testosterone.s, data_scaled_cor$avg_cortisol.s, na.rm = TR
UE)
```

```
##
##  Pearson's product-moment correlation
##
## data:  data_scaled_cor$testosterone.s and data_scaled_cor$avg_cortisol.s
## t = -2.4135e-15, df = 277, p-value = 1
## alternative hypothesis: true correlation is not equal to 0
## 95 percent confidence interval:
##  -0.1174317  0.1174317
## sample estimates:
##          cor
## -1.45014e-16
```

```
cor.test(data_scaled_cor$testosterone.s, data_scaled_cor$SOIR_behavior.s, na.rm = T
RUE)
```

```
##
##  Pearson's product-moment correlation
##
## data:  data_scaled_cor$testosterone.s and data_scaled_cor$SOIR_behavior.s
## t = -0.36063, df = 251, p-value = 0.7187
## alternative hypothesis: true correlation is not equal to 0
## 95 percent confidence interval:
##  -0.1456759  0.1008542
## sample estimates:
##         cor
## -0.02275679
```

```
cor.test(data_scaled_cor$testosterone.s, data_scaled_cor$SOIR_attitude.s, na.rm = T
RUE)
```

```
##
##  Pearson's product-moment correlation
##
## data:  data_scaled_cor$testosterone.s and data_scaled_cor$SOIR_attitude.s
## t = 0.13285, df = 264, p-value = 0.8944
## alternative hypothesis: true correlation is not equal to 0
## 95 percent confidence interval:
##  -0.1122059  0.1283214
## sample estimates:
##        cor
## 0.00817603
```

```
cor.test(data_scaled_cor$testosterone.s, data_scaled_cor$SOIR_desire.s, na.rm = TRU
E)
```

```
##
##  Pearson's product-moment correlation
##
## data:  data_scaled_cor$testosterone.s and data_scaled_cor$SOIR_desire.s
## t = -0.3949, df = 263, p-value = 0.6932
## alternative hypothesis: true correlation is not equal to 0
## 95 percent confidence interval:
##  -0.14441861  0.09643792
## sample estimates:
##         cor
## -0.02434361
```

```
cor.test(data_scaled_cor$testosterone.s, data_scaled_cor$SOIR_full.s, na.rm = TRUE)
```

```
##
##  Pearson's product-moment correlation
##
## data:  data_scaled_cor$testosterone.s and data_scaled_cor$SOIR_full.s
## t = -0.35177, df = 249, p-value = 0.7253
## alternative hypothesis: true correlation is not equal to 0
## 95 percent confidence interval:
##  -0.1457044  0.1018129
## sample estimates:
##         cor
## -0.02228729
```

```
cor.test(data_scaled_cor$testosterone.s, data_scaled_cor$SDI_solitary.s, na.rm = TR
UE)
```

```
##
##  Pearson's product-moment correlation
##
## data:  data_scaled_cor$testosterone.s and data_scaled_cor$SDI_solitary.s
## t = -0.6477, df = 262, p-value = 0.5177
## alternative hypothesis: true correlation is not equal to 0
## 95 percent confidence interval:
##  -0.15993827  0.08113537
## sample estimates:
##         cor
## -0.03998329
```

```
cor.test(data_scaled_cor$testosterone.s, data_scaled_cor$SDI_dyadic.s, na.rm = TRU
E)
```

```
##
##  Pearson's product-moment correlation
##
## data:  data_scaled_cor$testosterone.s and data_scaled_cor$SDI_dyadic.s
## t = 0.73656, df = 261, p-value = 0.462
## alternative hypothesis: true correlation is not equal to 0
## 95 percent confidence interval:
##  -0.07582955  0.16558934
## sample estimates:
##        cor
## 0.04554488
```

```
cor.test(data_scaled_cor$testosterone.s, data_scaled_cor$SDI_full.s, na.rm = TRUE)
```

```
##
##  Pearson's product-moment correlation
##
## data:  data_scaled_cor$testosterone.s and data_scaled_cor$SDI_full.s
## t = 0.13173, df = 258, p-value = 0.8953
## alternative hypothesis: true correlation is not equal to 0
## 95 percent confidence interval:
##  -0.1135663  0.1297249
## sample estimates:
##         cor
## 0.008200642
```

```
cor.test(data_scaled_cor$cortisol.s, data_scaled_cor$avg_testosterone.s, na.rm = TR
UE)
```

```
##
##  Pearson's product-moment correlation
##
## data:  data_scaled_cor$cortisol.s and data_scaled_cor$avg_testosterone.s
## t = -2.3533e-16, df = 280, p-value = 1
## alternative hypothesis: true correlation is not equal to 0
## 95 percent confidence interval:
##  -0.1168044  0.1168044
## sample estimates:
##           cor
## -1.406379e-17
```

```
cor.test(data_scaled_cor$cortisol.s, data_scaled_cor$avg_cortisol.s, na.rm = TRUE)
```

```
##
##  Pearson's product-moment correlation
##
## data:  data_scaled_cor$cortisol.s and data_scaled_cor$avg_cortisol.s
## t = -1.3019e-16, df = 280, p-value = 1
## alternative hypothesis: true correlation is not equal to 0
## 95 percent confidence interval:
##  -0.1168044  0.1168044
## sample estimates:
##           cor
## -7.780155e-18
```

```
cor.test(data_scaled_cor$cortisol.s, data_scaled_cor$SOIR_behavior.s, na.rm = TRUE)
```

```
##
##  Pearson's product-moment correlation
##
## data:  data_scaled_cor$cortisol.s and data_scaled_cor$SOIR_behavior.s
## t = -0.21093, df = 254, p-value = 0.8331
## alternative hypothesis: true correlation is not equal to 0
## 95 percent confidence interval:
##  -0.1356158  0.1095459
## sample estimates:
##        cor
## -0.0132338
```

```
cor.test(data_scaled_cor$cortisol.s, data_scaled_cor$SOIR_attitude.s, na.rm = TRUE)
```

```
##
##  Pearson's product-moment correlation
##
## data:  data_scaled_cor$cortisol.s and data_scaled_cor$SOIR_attitude.s
## t = 0.12048, df = 267, p-value = 0.9042
## alternative hypothesis: true correlation is not equal to 0
## 95 percent confidence interval:
##  -0.1123241  0.1268590
## sample estimates:
##         cor
## 0.007372913
```

```
cor.test(data_scaled_cor$cortisol.s, data_scaled_cor$SOIR_desire.s, na.rm = TRUE)
```

```
##
##  Pearson's product-moment correlation
##
## data:  data_scaled_cor$cortisol.s and data_scaled_cor$SOIR_desire.s
## t = -1.2197, df = 266, p-value = 0.2237
## alternative hypothesis: true correlation is not equal to 0
## 95 percent confidence interval:
##  -0.19267507  0.04565361
## sample estimates:
##         cor
## -0.07457555
```

```
cor.test(data_scaled_cor$cortisol.s, data_scaled_cor$SOIR_full.s, na.rm = TRUE)
```

```
##
##  Pearson's product-moment correlation
##
## data:  data_scaled_cor$cortisol.s and data_scaled_cor$SOIR_full.s
## t = -0.50687, df = 252, p-value = 0.6127
## alternative hypothesis: true correlation is not equal to 0
## 95 percent confidence interval:
##  -0.15439160  0.09153057
## sample estimates:
##         cor
## -0.03191351
```

```
cor.test(data_scaled_cor$cortisol.s, data_scaled_cor$SDI_solitary.s, na.rm = TRUE)
```

```
##
##  Pearson's product-moment correlation
##
## data:  data_scaled_cor$cortisol.s and data_scaled_cor$SDI_solitary.s
## t = -0.085842, df = 265, p-value = 0.9317
## alternative hypothesis: true correlation is not equal to 0
## 95 percent confidence interval:
##  -0.1252396  0.1148453
## sample estimates:
##          cor
## -0.005273162
```

```
cor.test(data_scaled_cor$cortisol.s, data_scaled_cor$SDI_dyadic.s, na.rm = TRUE)
```

```
##
##  Pearson's product-moment correlation
##
## data:  data_scaled_cor$cortisol.s and data_scaled_cor$SDI_dyadic.s
## t = -0.030853, df = 264, p-value = 0.9754
## alternative hypothesis: true correlation is not equal to 0
## 95 percent confidence interval:
##  -0.1221425  0.1183997
## sample estimates:
##          cor
## -0.001898856
```

```
cor.test(data_scaled_cor$cortisol.s, data_scaled_cor$SDI_full.s, na.rm = TRUE)
```

```
##
##  Pearson's product-moment correlation
##
## data:  data_scaled_cor$cortisol.s and data_scaled_cor$SDI_full.s
## t = -0.31883, df = 261, p-value = 0.7501
## alternative hypothesis: true correlation is not equal to 0
## 95 percent confidence interval:
##  -0.1403532  0.1014673
## sample estimates:
##         cor
## -0.01973151
```

```
cor.test(data_scaled_cor$avg_testosterone.s, data_scaled_cor$avg_cortisol.s, na.rm
= TRUE)
```

```
##
##  Pearson's product-moment correlation
##
## data:  data_scaled_cor$avg_testosterone.s and data_scaled_cor$avg_cortisol.s
## t = 10.671, df = 281, p-value < 2.2e-16
## alternative hypothesis: true correlation is not equal to 0
## 95 percent confidence interval:
##  0.4484837 0.6150856
## sample estimates:
##       cor
## 0.5370004
```

```
cor.test(data_scaled_cor$avg_testosterone.s, data_scaled_cor$SOIR_behavior.s, na.rm
= TRUE)
```

```
##
##  Pearson's product-moment correlation
##
## data:  data_scaled_cor$avg_testosterone.s and data_scaled_cor$SOIR_behavior.s
## t = 4.2491, df = 255, p-value = 3.011e-05
## alternative hypothesis: true correlation is not equal to 0
## 95 percent confidence interval:
##  0.1391598 0.3679299
## sample estimates:
##       cor
## 0.2571439
```

```
cor.test(data_scaled_cor$avg_testosterone.s, data_scaled_cor$SOIR_attitude.s, na.rm
= TRUE)
```

```
##
##  Pearson's product-moment correlation
##
## data:  data_scaled_cor$avg_testosterone.s and data_scaled_cor$SOIR_attitude.s
## t = 0.12325, df = 268, p-value = 0.902
## alternative hypothesis: true correlation is not equal to 0
## 95 percent confidence interval:
##  -0.1119479  0.1267906
## sample estimates:
##         cor
## 0.007528622
```

```
cor.test(data_scaled_cor$avg_testosterone.s, data_scaled_cor$SOIR_desire.s, na.rm =
TRUE)
```

```
##
##  Pearson's product-moment correlation
##
## data:  data_scaled_cor$avg_testosterone.s and data_scaled_cor$SOIR_desire.s
## t = 0.39554, df = 267, p-value = 0.6928
## alternative hypothesis: true correlation is not equal to 0
## 95 percent confidence interval:
##  -0.0956752  0.1433826
## sample estimates:
##        cor
## 0.02419963
```

```
cor.test(data_scaled_cor$avg_testosterone.s, data_scaled_cor$SOIR_full.s, na.rm = T
RUE)
```

```
##
##  Pearson's product-moment correlation
##
## data:  data_scaled_cor$avg_testosterone.s and data_scaled_cor$SOIR_full.s
## t = 1.8075, df = 253, p-value = 0.07188
## alternative hypothesis: true correlation is not equal to 0
## 95 percent confidence interval:
##  -0.01007466  0.23252510
## sample estimates:
##       cor
## 0.1129076
```

```
cor.test(data_scaled_cor$avg_testosterone.s, data_scaled_cor$SDI_solitary.s, na.rm
= TRUE)
```

```
##
##  Pearson's product-moment correlation
##
## data:  data_scaled_cor$avg_testosterone.s and data_scaled_cor$SDI_solitary.s
## t = -0.25436, df = 266, p-value = 0.7994
## alternative hypothesis: true correlation is not equal to 0
## 95 percent confidence interval:
##  -0.1351624  0.1044226
## sample estimates:
##        cor
## -0.0155937
```

```
cor.test(data_scaled_cor$avg_testosterone.s, data_scaled_cor$SDI_dyadic.s, na.rm =
TRUE)
```

```
##
##  Pearson's product-moment correlation
##
## data:  data_scaled_cor$avg_testosterone.s and data_scaled_cor$SDI_dyadic.s
## t = -2.7772, df = 265, p-value = 0.005875
## alternative hypothesis: true correlation is not equal to 0
## 95 percent confidence interval:
##  -0.28251218 -0.04911561
## sample estimates:
##        cor
## -0.1681698
```

```
cor.test(data_scaled_cor$avg_testosterone.s, data_scaled_cor$SDI_full.s, na.rm = TR
UE)
```

```
##
##  Pearson's product-moment correlation
##
## data:  data_scaled_cor$avg_testosterone.s and data_scaled_cor$SDI_full.s
## t = -2.5516, df = 262, p-value = 0.01129
## alternative hypothesis: true correlation is not equal to 0
## 95 percent confidence interval:
##  -0.27134433 -0.03566185
## sample estimates:
##        cor
## -0.1557184
```

```
cor.test(data_scaled_cor$avg_cortisol.s, data_scaled_cor$SOIR_behavior.s, na.rm = T
RUE)
```

```
##
##  Pearson's product-moment correlation
##
## data:  data_scaled_cor$avg_cortisol.s and data_scaled_cor$SOIR_behavior.s
## t = 2.5844, df = 255, p-value = 0.01031
## alternative hypothesis: true correlation is not equal to 0
## 95 percent confidence interval:
##  0.03814377 0.27671434
## sample estimates:
##       cor
## 0.1597609
```

```
cor.test(data_scaled_cor$avg_cortisol.s, data_scaled_cor$SOIR_attitude.s, na.rm = T
RUE)
```

```
##
##  Pearson's product-moment correlation
##
## data:  data_scaled_cor$avg_cortisol.s and data_scaled_cor$SOIR_attitude.s
## t = -2.2505, df = 268, p-value = 0.02523
## alternative hypothesis: true correlation is not equal to 0
## 95 percent confidence interval:
##  -0.25147732 -0.01709188
## sample estimates:
##        cor
## -0.1361899
```

```
cor.test(data_scaled_cor$avg_cortisol.s, data_scaled_cor$SOIR_desire.s, na.rm = TRU
E)
```

```
##
##  Pearson's product-moment correlation
##
## data:  data_scaled_cor$avg_cortisol.s and data_scaled_cor$SOIR_desire.s
## t = -1.8861, df = 267, p-value = 0.06036
## alternative hypothesis: true correlation is not equal to 0
## 95 percent confidence interval:
##  -0.231097055  0.004998057
## sample estimates:
##        cor
## -0.1146684
```

```
cor.test(data_scaled_cor$avg_cortisol.s, data_scaled_cor$SOIR_full.s, na.rm = TRUE)
```

```
##
##  Pearson's product-moment correlation
##
## data:  data_scaled_cor$avg_cortisol.s and data_scaled_cor$SOIR_full.s
## t = -0.97063, df = 253, p-value = 0.3327
## alternative hypothesis: true correlation is not equal to 0
## 95 percent confidence interval:
##  -0.18238750  0.06239984
## sample estimates:
##         cor
## -0.06090962
```

```
cor.test(data_scaled_cor$avg_cortisol.s, data_scaled_cor$SDI_solitary.s, na.rm = TR
UE)
```

```
##
##  Pearson's product-moment correlation
##
## data:  data_scaled_cor$avg_cortisol.s and data_scaled_cor$SDI_solitary.s
## t = -2.2007, df = 266, p-value = 0.02862
## alternative hypothesis: true correlation is not equal to 0
## 95 percent confidence interval:
##  -0.24954453 -0.01412684
## sample estimates:
##        cor
## -0.1337217
```

```
cor.test(data_scaled_cor$avg_cortisol.s, data_scaled_cor$SDI_dyadic.s, na.rm = TRU
E)
```

```
##
##  Pearson's product-moment correlation
##
## data:  data_scaled_cor$avg_cortisol.s and data_scaled_cor$SDI_dyadic.s
## t = -5.2133, df = 265, p-value = 3.739e-07
## alternative hypothesis: true correlation is not equal to 0
## 95 percent confidence interval:
##  -0.4100233 -0.1919725
## sample estimates:
##        cor
## -0.3049896
```

```
cor.test(data_scaled_cor$avg_cortisol.s, data_scaled_cor$SDI_full.s, na.rm = TRUE)
```

```
##
##  Pearson's product-moment correlation
##
## data:  data_scaled_cor$avg_cortisol.s and data_scaled_cor$SDI_full.s
## t = -4.7945, df = 262, p-value = 2.737e-06
## alternative hypothesis: true correlation is not equal to 0
## 95 percent confidence interval:
##  -0.3913197 -0.1690805
## sample estimates:
##        cor
## -0.2840101
```

```
cor.test(data_scaled_cor$SOIR_behavior.s, data_scaled_cor$SOIR_attitude.s, na.rm =
TRUE)
```

```
##
##  Pearson's product-moment correlation
##
## data:  data_scaled_cor$SOIR_behavior.s and data_scaled_cor$SOIR_attitude.s
## t = 7.0922, df = 254, p-value = 1.308e-11
## alternative hypothesis: true correlation is not equal to 0
## 95 percent confidence interval:
##  0.2988588 0.5040418
## sample estimates:
##      cor
## 0.406564
```

```
cor.test(data_scaled_cor$SOIR_behavior.s, data_scaled_cor$SOIR_desire.s, na.rm = TR
UE)
```

```
##
##  Pearson's product-moment correlation
##
## data:  data_scaled_cor$SOIR_behavior.s and data_scaled_cor$SOIR_desire.s
## t = 4.2598, df = 253, p-value = 2.888e-05
## alternative hypothesis: true correlation is not equal to 0
## 95 percent confidence interval:
##  0.1403119 0.3697867
## sample estimates:
##       cor
## 0.2586955
```

```
cor.test(data_scaled_cor$SOIR_behavior.s, data_scaled_cor$SOIR_full.s, na.rm = TRU
E)
```

```
##
##  Pearson's product-moment correlation
##
## data:  data_scaled_cor$SOIR_behavior.s and data_scaled_cor$SOIR_full.s
## t = 14.791, df = 253, p-value < 2.2e-16
## alternative hypothesis: true correlation is not equal to 0
## 95 percent confidence interval:
##  0.6090918 0.7417724
## sample estimates:
##       cor
## 0.6809817
```

```
cor.test(data_scaled_cor$SOIR_behavior.s, data_scaled_cor$SDI_solitary.s, na.rm = T
RUE)
```

```
##
##  Pearson's product-moment correlation
##
## data:  data_scaled_cor$SOIR_behavior.s and data_scaled_cor$SDI_solitary.s
## t = 1.6933, df = 253, p-value = 0.09164
## alternative hypothesis: true correlation is not equal to 0
## 95 percent confidence interval:
##  -0.01720965  0.22576349
## sample estimates:
##       cor
## 0.1058567
```

```
cor.test(data_scaled_cor$SOIR_behavior.s, data_scaled_cor$SDI_dyadic.s, na.rm = TRU
E)
```

```
##
##  Pearson's product-moment correlation
##
## data:  data_scaled_cor$SOIR_behavior.s and data_scaled_cor$SDI_dyadic.s
## t = 6.5424, df = 250, p-value = 3.398e-10
## alternative hypothesis: true correlation is not equal to 0
## 95 percent confidence interval:
##  0.2715965 0.4830861
## sample estimates:
##       cor
## 0.3823374
```

```
cor.test(data_scaled_cor$SOIR_behavior.s, data_scaled_cor$SDI_full.s, na.rm = TRUE)
```

```
##
##  Pearson's product-moment correlation
##
## data:  data_scaled_cor$SOIR_behavior.s and data_scaled_cor$SDI_full.s
## t = 5.8617, df = 249, p-value = 1.447e-08
## alternative hypothesis: true correlation is not equal to 0
## 95 percent confidence interval:
##  0.2345119 0.4525277
## sample estimates:
##       cor
## 0.3482198
```

```
cor.test(data_scaled_cor$SOIR_attitude.s, data_scaled_cor$SOIR_desire.s, na.rm = TR
UE)
```

```
##
##  Pearson's product-moment correlation
##
## data:  data_scaled_cor$SOIR_attitude.s and data_scaled_cor$SOIR_desire.s
## t = 7.343, df = 267, p-value = 2.531e-12
## alternative hypothesis: true correlation is not equal to 0
## 95 percent confidence interval:
##  0.3052648 0.5047513
## sample estimates:
##       cor
## 0.4098978
```

```
cor.test(data_scaled_cor$SOIR_attitude.s, data_scaled_cor$SOIR_full.s, na.rm = TRU
E)
```

```
##
##  Pearson's product-moment correlation
##
## data:  data_scaled_cor$SOIR_attitude.s and data_scaled_cor$SOIR_full.s
## t = 25.192, df = 253, p-value < 2.2e-16
## alternative hypothesis: true correlation is not equal to 0
## 95 percent confidence interval:
##  0.8064848 0.8772769
## sample estimates:
##       cor
## 0.8455574
```

```
cor.test(data_scaled_cor$SOIR_attitude.s, data_scaled_cor$SDI_solitary.s, na.rm = T
RUE)
```

```
##
##  Pearson's product-moment correlation
##
## data:  data_scaled_cor$SOIR_attitude.s and data_scaled_cor$SDI_solitary.s
## t = 5.5879, df = 265, p-value = 5.701e-08
## alternative hypothesis: true correlation is not equal to 0
## 95 percent confidence interval:
##  0.2129176 0.4280284
## sample estimates:
##       cor
## 0.3246649
```

```
cor.test(data_scaled_cor$SOIR_attitude.s, data_scaled_cor$SDI_dyadic.s, na.rm = TRU
E)
```

```
##
##  Pearson's product-moment correlation
##
## data:  data_scaled_cor$SOIR_attitude.s and data_scaled_cor$SDI_dyadic.s
## t = 8.2273, df = 262, p-value = 9.032e-15
## alternative hypothesis: true correlation is not equal to 0
## 95 percent confidence interval:
##  0.3516211 0.5440776
## sample estimates:
##       cor
## 0.4531133
```

```
cor.test(data_scaled_cor$SOIR_attitude.s, data_scaled_cor$SDI_full.s, na.rm = TRUE)
```

```
##
##  Pearson's product-moment correlation
##
## data:  data_scaled_cor$SOIR_attitude.s and data_scaled_cor$SDI_full.s
## t = 8.8247, df = 261, p-value < 2.2e-16
## alternative hypothesis: true correlation is not equal to 0
## 95 percent confidence interval:
##  0.3804843 0.5674336
## sample estimates:
##      cor
## 0.479379
```

```
cor.test(data_scaled_cor$SOIR_desire.s, data_scaled_cor$SOIR_full.s, na.rm = TRUE)
```

```
##
##  Pearson's product-moment correlation
##
## data:  data_scaled_cor$SOIR_desire.s and data_scaled_cor$SOIR_full.s
## t = 17.68, df = 253, p-value < 2.2e-16
## alternative hypothesis: true correlation is not equal to 0
## 95 percent confidence interval:
##  0.6829451 0.7937714
## sample estimates:
##       cor
## 0.7434188
```

```
cor.test(data_scaled_cor$SOIR_desire.s, data_scaled_cor$SDI_solitary.s, na.rm = TRU
E)
```

```
##
##  Pearson's product-moment correlation
##
## data:  data_scaled_cor$SOIR_desire.s and data_scaled_cor$SDI_solitary.s
## t = 4.3278, df = 264, p-value = 2.139e-05
## alternative hypothesis: true correlation is not equal to 0
## 95 percent confidence interval:
##  0.1414900 0.3663137
## sample estimates:
##       cor
## 0.2573816
```

```
cor.test(data_scaled_cor$SOIR_desire.s, data_scaled_cor$SDI_dyadic.s, na.rm = TRUE)
```

```
##
##  Pearson's product-moment correlation
##
## data:  data_scaled_cor$SOIR_desire.s and data_scaled_cor$SDI_dyadic.s
## t = 6.7277, df = 262, p-value = 1.079e-10
## alternative hypothesis: true correlation is not equal to 0
## 95 percent confidence interval:
##  0.2758621 0.4821911
## sample estimates:
##       cor
## 0.3838068
```

```
cor.test(data_scaled_cor$SOIR_desire.s, data_scaled_cor$SDI_full.s, na.rm = TRUE)
```

```
##
##  Pearson's product-moment correlation
##
## data:  data_scaled_cor$SOIR_desire.s and data_scaled_cor$SDI_full.s
## t = 6.7834, df = 261, p-value = 7.832e-11
## alternative hypothesis: true correlation is not equal to 0
## 95 percent confidence interval:
##  0.2792576 0.4853661
## sample estimates:
##       cor
## 0.3871376
```

```
cor.test(data_scaled_cor$SOIR_full.s, data_scaled_cor$SDI_solitary.s, na.rm = TRUE)
```

```
##
##  Pearson's product-moment correlation
##
## data:  data_scaled_cor$SOIR_full.s and data_scaled_cor$SDI_solitary.s
## t = 5.3572, df = 251, p-value = 1.912e-07
## alternative hypothesis: true correlation is not equal to 0
## 95 percent confidence interval:
##  0.2050980 0.4267912
## sample estimates:
##       cor
## 0.3203236
```

```
cor.test(data_scaled_cor$SOIR_full.s, data_scaled_cor$SDI_dyadic.s, na.rm = TRUE)
```

```
##
##  Pearson's product-moment correlation
##
## data:  data_scaled_cor$SOIR_full.s and data_scaled_cor$SDI_dyadic.s
## t = 10.046, df = 249, p-value < 2.2e-16
## alternative hypothesis: true correlation is not equal to 0
## 95 percent confidence interval:
##  0.4426464 0.6196477
## sample estimates:
##       cor
## 0.5370319
```

```
cor.test(data_scaled_cor$SOIR_full.s, data_scaled_cor$SDI_full.s, na.rm = TRUE)
```

```
##
##  Pearson's product-moment correlation
##
## data:  data_scaled_cor$SOIR_full.s and data_scaled_cor$SDI_full.s
## t = 10.094, df = 248, p-value < 2.2e-16
## alternative hypothesis: true correlation is not equal to 0
## 95 percent confidence interval:
##  0.4453974 0.6220628
## sample estimates:
##       cor
## 0.5396441
```

```
cor.test(data_scaled_cor$SDI_solitary.s, data_scaled_cor$SDI_dyadic.s, na.rm = TRU
E)
```

```
##
##  Pearson's product-moment correlation
##
## data:  data_scaled_cor$SDI_solitary.s and data_scaled_cor$SDI_dyadic.s
## t = 7.5575, df = 262, p-value = 6.9e-13
## alternative hypothesis: true correlation is not equal to 0
## 95 percent confidence interval:
##  0.3186101 0.5173664
## sample estimates:
##      cor
## 0.423064
```

```
cor.test(data_scaled_cor$SDI_solitary.s, data_scaled_cor$SDI_full.s, na.rm = TRUE)
```

```
##
##  Pearson's product-moment correlation
##
## data:  data_scaled_cor$SDI_solitary.s and data_scaled_cor$SDI_full.s
## t = 16.657, df = 262, p-value < 2.2e-16
## alternative hypothesis: true correlation is not equal to 0
## 95 percent confidence interval:
##  0.6529797 0.7711315
## sample estimates:
##       cor
## 0.7171705
```

```
cor.test(data_scaled_cor$SDI_dyadic.s, data_scaled_cor$SDI_full.s, na.rm = TRUE)
```

```
##
##  Pearson's product-moment correlation
##
## data:  data_scaled_cor$SDI_dyadic.s and data_scaled_cor$SDI_full.s
## t = 36.92, df = 262, p-value < 2.2e-16
## alternative hypothesis: true correlation is not equal to 0
## 95 percent confidence interval:
##  0.8939647 0.9333742
## sample estimates:
##       cor
## 0.9158481
```

```
###Repeating analyses from Puts et al., 2015

#First: Cross sectional
Putsdata = data_scaled %>%
  filter(session == "1")

Putsdata$sociosexpsych <- rowMeans(Putsdata[,c(15:20)])

Putsmodel1 <- lm(testosterone.s ~ sociosexpsych + behavior1, data = Putsdata)
summary.Putsmodel1 <- summary(Putsmodel1)

summary.Putsmodel1$coefficients %>%
  as.data.frame() %>%
  rownames_to_column(var = "Effect") %>%
  mutate_if(is.numeric, round, 3) %>%
  rename(p = `Pr(>|t|)`)
```

```
##          Effect Estimate Std. Error t value     p
## 1   (Intercept)    0.123      0.081   1.530 0.132
## 2 sociosexpsych   -0.022      0.023  -0.931 0.356
## 3     behavior1   -0.023      0.023  -0.985 0.329
```

```
#Second: Longitudinal

data_scaled$sociosexpsych <- rowMeans(data_scaled[,c(15:20)])



Putsmodel2 <- lmer(testosterone.s ~ 1 + sociosexpsych + behavior1 +
                        (sociosexpsych + behavior1 || hm_id), data = data_scaled, R
EML = FALSE)
```

```
## boundary (singular) fit: see ?isSingular
```

```r
summary.Putsmodel2 <- summary(Putsmodel2)

summary.Putsmodel2$coefficients %>%
  as.data.frame() %>%
  rownames_to_column(var = "Effect") %>%
  mutate_if(is.numeric, round, 3) %>%
  rename(p = `Pr(>|t|)`)
```

```
##          Effect Estimate Std. Error  df t value     p
## 1   (Intercept)    0.009      0.033 251   0.284 0.777
## 2 sociosexpsych   -0.002      0.009 251  -0.169 0.866
## 3     behavior1   -0.002      0.009 251  -0.207 0.836
```

```r
#analyses for men's relationship status

#contrast code relationship status (-1 = single, 1 = partnered)
library(car)
```

```
## Loading required package: carData
```

```
## Registered S3 methods overwritten by 'car':
##   method                          from
##   influence.merMod                lme4
##   cooks.distance.influence.merMod lme4
##   dfbeta.influence.merMod         lme4
##   dfbetas.influence.merMod        lme4
```

```
##
## Attaching package: 'car'
```

```
## The following object is masked from 'package:psych':
##
##     logit
```

```
## The following object is masked from 'package:purrr':
##
##     some
```

```
## The following object is masked from 'package:dplyr':
##
##     recode
```

```
data_scaled$relationship <- car::recode(data_scaled$relationship, "0 = -1")

#Following Edelstein et al., 2011: Investigating associations between testosterone
and SOI-R subscales interacting with relationship status

#first between-subjects
Edelsteinmodel1 <- lm(avg_testosterone.s ~ SOIR_desire * relationship + SOIR_behavi
or * relationship + SOIR_attitude * relationship, data = Putsdata)
summary.Edelsteinmodel1 <- summary(Edelsteinmodel1)

summary.Edelsteinmodel1$coefficients %>%
  as.data.frame() %>%
  rownames_to_column(var = "Effect") %>%
  mutate_if(is.numeric, round, 3) %>%
  rename(p = `Pr(>|t|)`)
```

```
##                        Effect Estimate Std. Error t value     p
## 1                (Intercept)    0.053      0.149   0.355 0.724
## 2                SOIR_desire    0.000      0.043   0.006 0.995
## 3              relationship1   -0.480      0.220  -2.184 0.034
## 4              SOIR_behavior    0.054      0.065   0.836 0.407
## 5              SOIR_attitude   -0.034      0.041  -0.836 0.407
## 6   SOIR_desire:relationship1    0.029      0.063   0.451 0.654
## 7 relationship1:SOIR_behavior    0.052      0.077   0.680 0.500
## 8 relationship1:SOIR_attitude    0.048      0.056   0.846 0.402
```

```
#second within-subjects
Edelsteinmodel2 <- lmer(testosterone.s ~ 1 + SOIR_desire * relationship + SOIR_beha
vior * relationship + SOIR_attitude * relationship +
                        (SOIR_desire + SOIR_behavior + SOIR_attitude + relationship
|| hm_id), data = data_scaled, REML = FALSE)
```

```
## boundary (singular) fit: see ?isSingular
```

```
summary.Edelsteinmodel2 <- summary(Edelsteinmodel2)

summary.Edelsteinmodel2$coefficients %>%
  as.data.frame() %>%
  rownames_to_column(var = "Effect") %>%
  mutate_if(is.numeric, round, 3) %>%
  rename(p = `Pr(>|t|)`)
```

```
##                         Effect Estimate Std. Error  df t value     p
## 1                  (Intercept)    0.023      0.044 251   0.522 0.602
## 2                  SOIR_desire   -0.004      0.013 251  -0.322 0.748
## 3                relationship1   -0.017      0.065 251  -0.266 0.790
## 4                SOIR_behavior    0.006      0.019 251   0.298 0.766
## 5                SOIR_attitude   -0.006      0.012 251  -0.468 0.640
## 6   SOIR_desire:relationship1   -0.003      0.019 251  -0.139 0.890
## 7 relationship1:SOIR_behavior   -0.014      0.024 251  -0.582 0.561
## 8 relationship1:SOIR_attitude    0.015      0.016 251   0.907 0.365
```

As requested in the review process: Repeat all main models including relationship status as a covariate (exploratory analyses part 3)

```
##full scale sdi
SDI_full_model_rel <- lmer(SDI_full ~ 1 + testosterone.s * cortisol.s +
                        avg_testosterone.s * avg_cortisol.s + relationship+
                        (testosterone.s * cortisol.s + relationship || hm_id),
                     data = data_scaled, REML = FALSE)
```

```
## Warning in checkConv(attr(opt, "derivs"), opt$par, ctrl =
## control$checkConv, : unable to evaluate scaled gradient
```

```
## Warning in checkConv(attr(opt, "derivs"), opt$par, ctrl =
## control$checkConv, : Model failed to converge: degenerate Hessian with 2
## negative eigenvalues
```

```
## Warning: Model failed to converge with 2 negative eigenvalues: -3.5e-03
## -4.4e-03
```

```
summary.SDI_full_model_rel <- summary(SDI_full_model_rel)

summary.SDI_full_model_rel$coefficients %>%
  as.data.frame() %>%
  rownames_to_column(var = "Effect") %>%
  mutate_if(is.numeric, round, 3) %>%
  rename(p = `Pr(>|t|)`)
```

```
##                              Effect Estimate Std. Error     df t value
## 1                       (Intercept)   56.009      3.440 28.938  16.281
## 2                    testosterone.s    1.250      2.786 30.606   0.449
## 3                        cortisol.s   -2.002      2.246 11.273  -0.891
## 4                avg_testosterone.s   13.492     10.193 53.598   1.324
## 5                    avg_cortisol.s  -12.420     12.856 59.056  -0.966
## 6                     relationship1   11.610      3.971 46.598   2.924
## 7         testosterone.s:cortisol.s   26.189     17.904 61.893   1.463
## 8 avg_testosterone.s:avg_cortisol.s  -80.897     45.777 56.917  -1.767
##       p
## 1 0.000
## 2 0.657
## 3 0.392
## 4 0.191
## 5 0.338
## 6 0.005
## 7 0.149
## 8 0.083
```

```
#sdi solitary
SDI_solitary_model_rel <- lmer(SDI_solitary ~ 1 + testosterone.s * cortisol.s +
                        avg_testosterone.s * avg_cortisol.s + relationship+
                        (testosterone.s * cortisol.s + relationship|| hm_id),
                     data = data_scaled, REML = FALSE)
```

```
## boundary (singular) fit: see ?isSingular
```

```
summary.SDI_solitary_model_rel <- summary(SDI_solitary_model_rel)

summary.SDI_solitary_model_rel$coefficients %>%
  as.data.frame() %>%
  rownames_to_column(var = "Effect") %>%
  mutate_if(is.numeric, round, 3) %>%
  rename(p = `Pr(>|t|)`)
```

```
##                               Effect Estimate Std. Error      df t value
## 1                        (Intercept)   10.807      0.855  28.107  12.643
## 2                     testosterone.s   -1.646      0.928 202.486  -1.774
## 3                         cortisol.s    0.403      0.772 203.583   0.522
## 4                 avg_testosterone.s    4.526      3.614  58.626   1.252
## 5                     avg_cortisol.s   -4.174      4.362  55.651  -0.957
## 6                      relationship1    0.990      1.242  59.527   0.797
## 7          testosterone.s:cortisol.s    2.214      6.271 207.306   0.353
## 8 avg_testosterone.s:avg_cortisol.s  -28.136     15.625  55.031  -1.801
##       p
## 1 0.000
## 2 0.078
## 3 0.602
## 4 0.215
## 5 0.343
## 6 0.428
## 7 0.724
## 8 0.077
```

```
#sdi dyadic
SDI_dyadic_model_rel <- lmer(SDI_dyadic ~ 1 + testosterone.s * cortisol.s +
                        avg_testosterone.s * avg_cortisol.s + relationship+
                        (testosterone.s * cortisol.s + relationship|| hm_id),
                     data = data_scaled, REML = FALSE)
```

```
## Warning in checkConv(attr(opt, "derivs"), opt$par, ctrl =
## control$checkConv, : Model failed to converge with max|grad| = 0.00657013
## (tol = 0.002, component 1)
```

```
summary.SDI_dyadic_model_rel <- summary(SDI_dyadic_model_rel)

summary.SDI_dyadic_model_rel$coefficients %>%
  as.data.frame() %>%
  rownames_to_column(var = "Effect") %>%
  mutate_if(is.numeric, round, 3) %>%
  rename(p = `Pr(>|t|)`)
```

```
##                              Effect Estimate Std. Error      df t value
## 1                       (Intercept)   35.282      2.195  28.450  16.074
## 2                    testosterone.s    3.954      2.339  30.475   1.690
## 3                        cortisol.s   -1.113      1.669 190.173  -0.667
## 4                avg_testosterone.s    6.891      5.688  49.269   1.212
## 5                    avg_cortisol.s   -5.888      7.282  56.106  -0.809
## 6                     relationship1    7.324      2.431  41.448   3.013
## 7         testosterone.s:cortisol.s   20.042     13.499 208.447   1.485
## 8 avg_testosterone.s:avg_cortisol.s  -21.968     25.765  52.606  -0.853
##       p
## 1 0.000
## 2 0.101
## 3 0.506
## 4 0.231
## 5 0.422
## 6 0.004
## 7 0.139
## 8 0.398
```

Results for LMEM Analysis for SOI_R

```
#SOIR full scale
SOIR_full_model_rel <- lmer(SOIR_full ~ 1 + testosterone.s * cortisol.s +
                        avg_testosterone.s * avg_cortisol.s + relationship+
                        (testosterone.s * cortisol.s + relationship|| hm_id),
                     data = data_scaled, REML = FALSE)
```

```
## Warning in checkConv(attr(opt, "derivs"), opt$par, ctrl =
## control$checkConv, : Model failed to converge with max|grad| = 0.00951461
## (tol = 0.002, component 1)
```

```
summary.SOIR_full_model_rel <- summary(SOIR_full_model_rel)

summary.SOIR_full_model_rel$coefficients %>%
  as.data.frame() %>%
  rownames_to_column(var = "Effect") %>%
  mutate_if(is.numeric, round, 3) %>%
  rename(p = `Pr(>|t|)`)
```

```
##                              Effect Estimate Std. Error     df t value
## 1                       (Intercept)    3.059      0.147 25.217  20.824
## 2                    testosterone.s   -0.050      0.131 37.999  -0.380
## 3                        cortisol.s   -0.131      0.119 28.408  -1.097
## 4                avg_testosterone.s    1.120      0.521 56.981   2.150
## 5                    avg_cortisol.s   -0.176      0.644 58.997  -0.273
## 6                     relationship1    0.080      0.188 52.542   0.425
## 7         testosterone.s:cortisol.s    0.663      0.938 19.932   0.707
## 8 avg_testosterone.s:avg_cortisol.s   -5.732      2.282 57.717  -2.512
##       p
## 1 0.000
## 2 0.706
## 3 0.282
## 4 0.036
## 5 0.786
## 6 0.672
## 7 0.488
## 8 0.015
```

```
#SOIR behavior
SOIR_behavior_model_rel <- lmer(SOIR_behavior ~ 1 + testosterone.s * cortisol.s +
                        avg_testosterone.s * avg_cortisol.s + relationship+
                        (testosterone.s * cortisol.s+ relationship || hm_id),
                     data = data_scaled, REML = FALSE)
```

```
## boundary (singular) fit: see ?isSingular
```

```
## Warning: Model failed to converge with 1 negative eigenvalue: -8.5e-03
```

```
summary.SOIR_behavior_model_rel <- summary(SOIR_behavior_model_rel)

summary.SOIR_behavior_model_rel$coefficients %>%
  as.data.frame() %>%
  rownames_to_column(var = "Effect") %>%
  mutate_if(is.numeric, round, 3) %>%
  rename(p = `Pr(>|t|)`)
```

```
##                              Effect Estimate Std. Error      df t value
## 1                       (Intercept)    1.945      0.165  22.814  11.821
## 2                    testosterone.s   -0.144      0.139 192.970  -1.041
## 3                        cortisol.s   -0.054      0.115 193.999  -0.465
## 4                avg_testosterone.s    1.242      0.599  56.517   2.074
## 5                    avg_cortisol.s    0.354      0.740  59.053   0.478
## 6                     relationship1    0.493      0.213  53.547   2.311
## 7         testosterone.s:cortisol.s    1.644      0.954 197.284   1.723
## 8 avg_testosterone.s:avg_cortisol.s    0.157      2.626  57.677   0.060
##       p
## 1 0.000
## 2 0.299
## 3 0.642
## 4 0.043
## 5 0.634
## 6 0.025
## 7 0.086
## 8 0.953
```

```
#SOIR attitude
SOIR_attitude_model_rel <- lmer(SOIR_attitude ~ 1 + testosterone.s * cortisol.s +
                        avg_testosterone.s * avg_cortisol.s + relationship+
                        (testosterone.s * cortisol.s + relationship|| hm_id),
                     data = data_scaled, REML = FALSE)
```

```
## Warning in checkConv(attr(opt, "derivs"), opt$par, ctrl =
## control$checkConv, : unable to evaluate scaled gradient
```

```
## Warning in checkConv(attr(opt, "derivs"), opt$par, ctrl =
## control$checkConv, : Model failed to converge: degenerate Hessian with 2
## negative eigenvalues
```

```
## Warning: Model failed to converge with 2 negative eigenvalues: -4.5e-04
## -1.1e-03
```

```
summary.SOIR_attitude_model_rel <- summary(SOIR_attitude_model_rel)

summary.SOIR_attitude_model_rel$coefficients %>%
  as.data.frame() %>%
  rownames_to_column(var = "Effect") %>%
  mutate_if(is.numeric, round, 3) %>%
  rename(p = `Pr(>|t|)`)
```

```
##                               Effect Estimate Std. Error     df t value
## 1                        (Intercept)    3.706      0.190 28.676  19.516
## 2                     testosterone.s    0.115      0.255 35.800   0.449
## 3                         cortisol.s    0.094      0.215 23.139   0.439
## 4                 avg_testosterone.s    1.336      0.764 61.254   1.749
## 5                     avg_cortisol.s   -0.277      0.928 60.276  -0.299
## 6                      relationship1    0.110      0.262 59.804   0.419
## 7          testosterone.s:cortisol.s   -1.501      1.541 10.016  -0.974
## 8 avg_testosterone.s:avg_cortisol.s  -11.928      3.317 58.994  -3.596
##       p
## 1 0.000
## 2 0.656
## 3 0.665
## 4 0.085
## 5 0.766
## 6 0.677
## 7 0.353
## 8 0.001
```

```
#SOIR desire
SOIR_desire_model_rel <- lmer(SOIR_desire ~ 1 + testosterone.s * cortisol.s +
                        avg_testosterone.s * avg_cortisol.s + relationship+
                        (testosterone.s * cortisol.s + relationship|| hm_id),
                     data = data_scaled, REML = FALSE)
```

```
## Warning in checkConv(attr(opt, "derivs"), opt$par, ctrl =
## control$checkConv, : Model failed to converge with max|grad| = 0.00722477
## (tol = 0.002, component 1)
```

```
summary.SOIR_desire_model_rel <- summary(SOIR_desire_model_rel)

summary.SOIR_desire_model_rel$coefficients %>%
  as.data.frame() %>%
  rownames_to_column(var = "Effect") %>%
  mutate_if(is.numeric, round, 3) %>%
  rename(p = `Pr(>|t|)`)
```

```
##                                Effect Estimate Std. Error      df t value
## 1                         (Intercept)    3.551      0.157  29.109  22.595
## 2                      testosterone.s   -0.002      0.207 202.291  -0.010
## 3                          cortisol.s   -0.489      0.237  45.462  -2.066
## 4                  avg_testosterone.s    0.647      0.640  61.451   1.011
## 5                      avg_cortisol.s   -0.756      0.776  60.193  -0.974
## 6                       relationship1   -0.361      0.218  59.709  -1.656
## 7           testosterone.s:cortisol.s    1.839      1.682  18.469   1.093
## 8 avg_testosterone.s:avg_cortisol.s   -5.887      2.767  58.271  -2.128
##       p
## 1 0.000
## 2 0.992
## 3 0.045
## 4 0.316
## 5 0.334
## 6 0.103
## 7 0.288
## 8 0.038
```
